# Mechanisms driving the antibacterial and antibiofilm properties of Hp1404 and its analogue peptides against multidrug-resistant *Pseudomonas aeruginosa*

**DOI:** 10.1038/s41598-018-19434-7

**Published:** 2018-01-29

**Authors:** Min Kyung Kim, Hee Kyoung Kang, Su Jin Ko, Min Ji Hong, Jeong Kyu Bang, Chang Ho Seo, Yoonkyung Park

**Affiliations:** 1 Research Center for proteineous Materials (RCPM), Chosun University, Kwangju, Republic of Korea; 2 Department of Biotechnology and BK21-Plus Research Team for Bioactive Control Technology, Chosun University, Kwangju, Republic of Korea; 3 Division of Magnetic Resonance, Korea Basic Science Institute, Ochang, Chung-Buk, 363-883 Republic of Korea; 4 Department of Bioinformatics, Kongju National University, Kongju, 314-701 South Korea

## Abstract

Hp1404, identified from the venom of the scorpion *Heterometrus petersii*, displays antimicrobial activity with cytotoxicity. Several synthetic peptides were designed based on the parent peptide Hp1404 to reduce cytotoxicity and improve activity (deletion of glycine and phenylalanine, substitution with leucine and lysine). The analogue peptides generated comprised 12 amino acids and displayed amphipathic α-helical structures, with higher hydrophobic moments and net positive charge than those of the Hp1404. The analogues showed less hemolytic and toxic effects toward mammalian cells than the Hp1404, especially Hp1404-T1e, which exhibited particularly potent antibacterial and antibiofilm activities against multidrug-resistant *Pseudomonas aeruginosa* (MRPA) strains. The analogue peptide Hp1404-T1e was more stable against salt and trypsin than the Hp1404. Hp1404’s mechanism of action involves binding to lipopolysaccharide (LPS), thereby killing bacteria through membrane disruption. Hp1404-T1e kills bacteria more rapidly than Hp1404 and not only seems to bind more strongly to LPS but may also be able to enter bacterial cells and interact with their DNA. Additionally, Hp1404-T1e can effectively kill bacteria *in vivo*. The results of this study indicate that Hp1404-T1e not only displays antimicrobial activity, but is also functional in physiological conditions, confirming its potential use as an effective therapeutic agent against MRPA.

## Introduction

Since their discovery, antibiotics have been widely used as therapeutic agents against infectious diseases. However, their indiscriminate use has led to the rapid emergence of increasingly resistant strains. The best example is penicillin, since its introduction for clinical use in the 1940s, numerous bacterial strains have developed resistance to it^1^. Nowadays, the list of antibiotic-resistant bacteria includes vancomycin-resistant *Staphylococcus aureus* (VRSA), methicillin-resistant *Staphylococcus aureus* (MRSA), multidrug-resistant *Pseudomonas aeruginosa* (MRPA)^2^, multidrug-resistant *Acinetobacter baumannii* (MRAB), among others. Recently, carbapenem-resistant *Enterobacteriaceae* (CRE) have also become an issue. As the emergence of multidrug-resistant microbes has grown into a global problem, significant research efforts are currently being channelled into the development of alternative antibiotics. Antimicrobial peptides (AMPs) are promising candidates, as their use does not lead to the development of resistance^3,4^.

AMPs are important components of the innate immune system; they have been isolated from a variety of organisms such as bacteria, insects^5^, reptiles^6^, mammals^5^, and plants^7^, while they can also be chemically synthesized^8^. AMPs can be used against a broad spectrum of pathogenic agents such as gram-negative and gram-positive bacteria, fungi, parasites, and viruses^9^. They are small biological molecules consisting of 12–50 amino acid residues. Moreover, AMPs have a net charge ranging from +2 to +7 owing to a surplus of basic amino acids (arginine, lysine, and histidine), and display amphipathic properties^10^. Based on their secondary structure, which plays a role in determining their target-cell specificity, AMPs are classified as α-helical, β-sheet, loop, or extended^11,12^. With respect to their mechanism of action, the ability of AMPs to kill bacteria through membrane permeabilization has been well established. Three different models have been proposed: the ‘toroidal’, the ‘barrel-stave’, and the ‘carpet’ model. However, there are also non-membrane-permeabilizing modes of action; some AMPs enter the bacterial cytoplasm and cause damage by interacting with intracellular targets such as DNA, RNA, and proteins^13,14^.

Bacterial biofilms are formed by closely associated microbial cells. They were discovered in patients with difficult-to-treat microbial infections. Biofilms can form on or in various medical devices such as urinary catheters, prosthetic heart valves, central venous catheters, vascular prosthesis^15^, intravenous catheters^16^, contact lenses^17^, and cardiac pacemakers^18^. Bacteria growing in biofilms are far more resistant (up to 1000 times) to antibiotics than planktonic cells of the same organism. An example of a biofilm-forming bacterium is *Pseudomonas aeruginosa*, an opportunistic human pathogen that infects eyes, lungs, and other organs^19^.

In a previous study, Hp1404 was purified from the venomous gland of the scorpion *Heterometrus petersii*. It consists of 14 amino acids and exhibits potent activity against gram-positive bacteria, including *Staphylococcus aureus*. Moreover, it is not toxic to mammalian cells at concentrations below 50 µM^20^.

In this study, we designed a set of 12 amino acid-analogues of Hp1404. Based on a previous report^21^, we tried to decrease toxicity by removing Glycine (Gly, G) and Phenylalanine (Phe, F) residues, while adding Lysine (Lys, K) and Leucine (Leu, L) residues. Hp1404 and its analogue peptides were examined for activity against gram-negative and gram-positive bacteria, including antibiotic-resistant strains of *Pseudomonas aeruginosa*. Among them, Hp1404-T1e exhibited potent antibacterial and antibiofilm activities when tested against both drug-susceptible and multidrug-resistant strains of *P. aeruginosa*. Thus, we believe that this newly designed peptide could be developed into an effective therapeutic agent.

## Results

### Synthetic peptide design and characterization

Hp1404 was isolated from the venomous gland of the scorpion *Heterometrus petersii*^20^. The analogue peptides tested in this study were designed by truncating G and F residues, while K and L residues were added^22^. The amino acid sequence, observed and calculated molecular weight, hydrophobic moment, and retention time of each peptide are summarized in Table 1. The hydrophobic moment values of Hp1404-T1, Hp1404-T1a, Hp1404-T1b, Hp1404-T1c, Hp1404-T1d, and Hp1404-T1e were 0.699, 0.761, 0.755, 0.784, 0.768, and 0.831, respectively, thus higher than the ones recorded for the parent peptide (0.677). The net charge values of the analogue peptides ranged from +1 to +6; all but Hp1404-T1 (+1) were more positively charged than Hp1404 (+1).

**Table 1:** Sequence and physicochemical parameters of Hp1404 and its synthetic analogue peptides.

Peptide	Sequence	Retention Time^a^	Observed	Calculated	µH^b^	Net charge
Hp1404	GILGKLWEGVKSIF-NH_2_	36.251	1545.9	1545.7	0.67754	+1
Hp1404-T1	ILGKLWEGVKSI-NH_2_	23.176	1341.7	1342.2	0.69906	+1
Hp1404-T1a	IL***K***KLWEGVKSI-NH_2_	20.756	1412.8	1413.5	0.76138	+2
Hp1404-T1b	IL***K***KL***L***EGVKSI-NH_2_	20.875	1339.7	1340.2	0.75568	+2
Hp1404-T1c	IL***K***KL***LK***GVKSI-NH_2_	17.643	1338.8	1338.0	0.78476	+4
Hp1404-T1d	IL***K***KL***LKK***VKSI-NH_2_	17.741	1409.9	1409.8	0.76840	+5
Hp1404-T1e	IL***K***KL***LKK***VK***K***I-NH_2_	16.002	1450.9	1451.3	0.83187	+6

^a^Mean retention time (min) in reversed-phase high performance liquid chromatography (RP-HPLC). ^b^Hydrophobic moment (µH) was calculated using the HeliQuest site.

**Figure 1:**
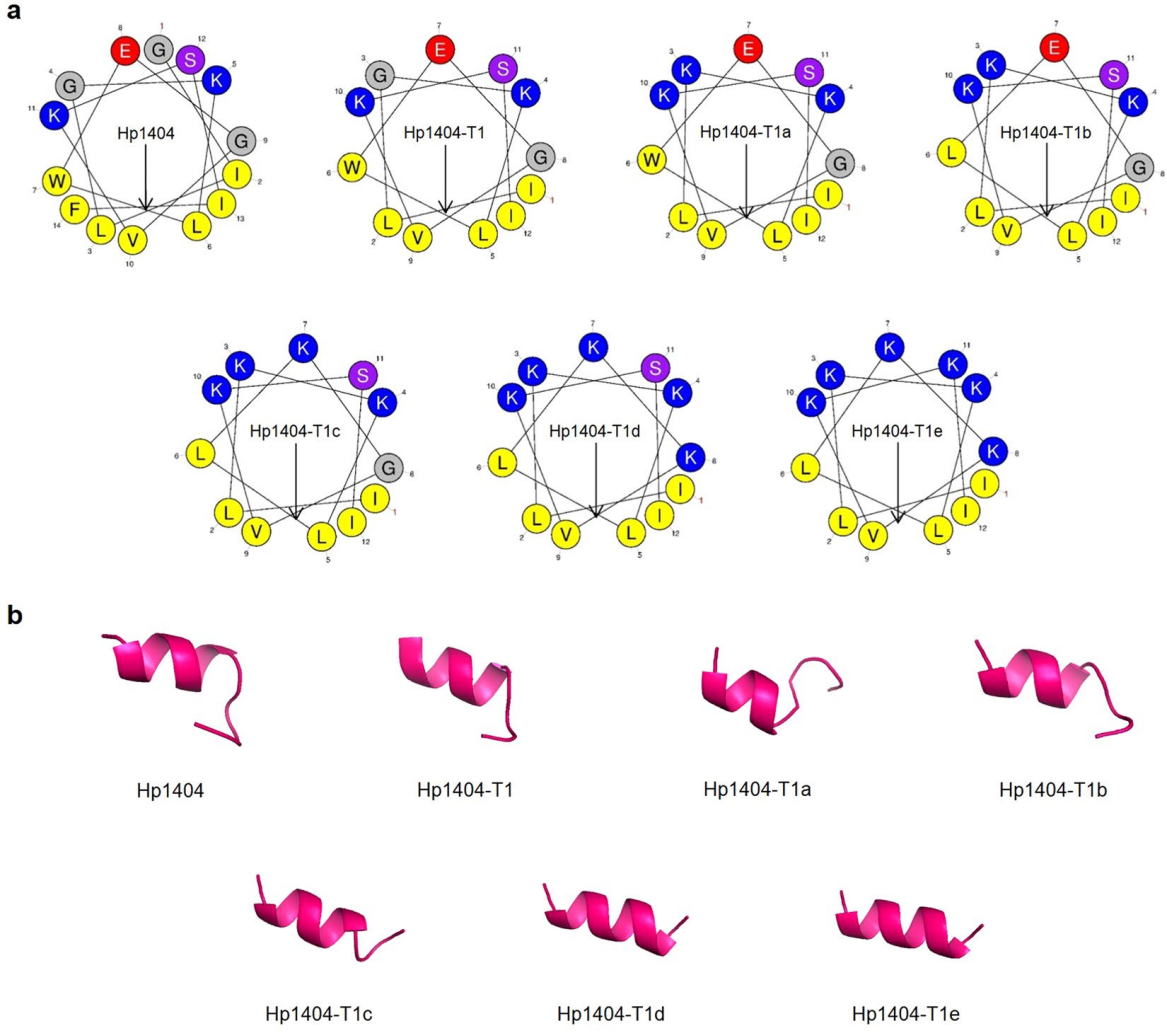
Structural analyses of Hp1404 and its analogue peptides. (**a**) Helical wheel projection of Hp1404 and its analogue peptides. The HeliQuest site and the Mobyle@RPBS portal were used for the construction of diagrams and projections, respectively. The hydrophobic residues are represented in yellow, negatively charged residues are red, positively charged residues are blue, and the particular polar residue is violet. The arrows represent the helical hydrophobic moment. (**b**) Three-dimensional structure simulations of the peptides are depicted in the ribbon diagrams.

Figure 1 displays helical wheel diagrams and three-dimensional structure projections for the parent peptide and its analogues. Hp1404 and its analogue peptides show amphipathic α-helix conformations with hydrophobic and hydrophilic residues located on opposite sides of the α-helix. Compared to Hp1404, analogue peptides appeared to display stronger amphipathic α-helical structures. Successful peptide synthesis was confirmed by RP-HPLC using C18 columns (Fig. S1), while the molecular weight of each peptide was determined through MALDI-TOF/MS (Fig. S2).

### Antibacterial activity

We studied the antibacterial activity of the peptides against *P. aeruginosa*. As shown in Table 2, the parent peptide exhibited broad-spectrum antimicrobial activity, as it was effective against *pseudomonas aeruginosa* strains with minimum inhibitory concentration (MIC) values ranging from 3.13 to 12.5 µM. In contrast, Hp1404-T1, Hp1404-T1a, and Hp1404-T1b had no activity at 25 µM against any of them. Three other analogues, Hp1404-T1c, Hp1404-T1d, and Hp1404-T1e, showed antimicrobial activities when tested against *P. aeruginosa* strains. In the case of ciprofloxacin, which is an antibiotic for the treatment of *P. aeruginosa*^23^, activity was not observed at a concentration of 400 µM. The peptides that were active against the drug-susceptible strain also proved effective against the resistant strains. Notably, Hp1404-T1e displayed MIC values lower than those of the parent peptide and the other analogues against both drug-susceptible and multidrug-resistant strains of *P. aeruginosa*. We conclude that Hp1404-T1e has potent antibacterial activity against *P. aeruginosa*.

**Table 2:** Antimicrobial activity of peptides against *Pseudomonas aeruginosa* and MRPA strains.

Peptides	MIC (µM)									
	Hp1404	Hp1404 -T1	Hp1404 -T1a	Hp1404 -T1b	Hp1404 -T1c	Hp1404 -T1d	Hp1404 -T1e	Buforin2	Melittin	Ciprofloxacin
*P. aeruginosa* ATCC 27853	12.5	>25	>25	>25	3.13	1.56	1.56	25	3.13	1.56
*P. aeruginosa* 138	12.5	>25	>25	>25	25	12.5	6.25	12.5	3.13	>400
*P. aeruginosa* 431	12.5	>25	25	>25	12.5	3.13	3.13	6.25	3.13	>400
*P. aeruginosa* 434	12.5	>25	25	25	6.25	3.13	3.13	6.25	3.13	>400
*P. aeruginosa* 557	6.25	>25	>25	>25	25	3.13	3.13	6.25	1.56	>400
*P. aeruginosa* 559	25	>25	>25	>25	25	12.5	6.25	12.5	3.13	>400
*P. aeruginosa* 778	12.5	>25	>25	>25	6.25	3.13	1.56	12.5	3.13	>400
*P. aeruginosa* 1034	6.25	>25	>25	>25	6.25	3.13	1.56	6.25	1.56	>400
*P. aeruginosa* 1162	12.5	>25	>25	>25	3.13	1.56	1.56	12.5	3.13	>400
*P. aeruginosa* 3290	12.5	>25	>25	>25	>25	6.25	6.25	6.25	3.13	>400
*P. aeruginosa* 3399	6.25	>25	25	>25	3.13	1.56	0.78	12.5	3.13	>400
*P. aeruginosa* 3543	6.25	>25	>25	>25	>25	25	12.5	12.5	3.13	400
*P. aeruginosa* 3592	6.25	>25	12.5	25	6.25	3.13	1.56	3.13	3.13	>400
*P. aeruginosa* 3904	6.25	>25	25	>25	6.25	6.25	3.13	6.25	3.13	400
*P. aeruginosa* 4007	25	>25	>25	>25	12.5	6.25	3.13	12.5	3.13	>400
*P*. aeruginosa 4319	12.5	>25	>25	>25	6.25	3.13	3.13	12.5	3.13	>400
*P. aeruginosa* 4891	6.25	>25	25	>25	6.25	3.13	1.56	>25	1.56	>400
*P. aeruginosa* 5018	12.5	>25	>25	>25	3.13	1.56	1.56	12.5	3.13	>400
*P. aeruginosa* 671973	3.13	>25	25	>25	6.25	1.56	1.56	3.13	1.56	100

Multidrug-resistant *pseudomonas aeruginosa* strains were obtained from Chonnam National University Hospital, Korea.

### Hemolytic and cytotoxic activities of synthetic peptides

**Figure 2:**
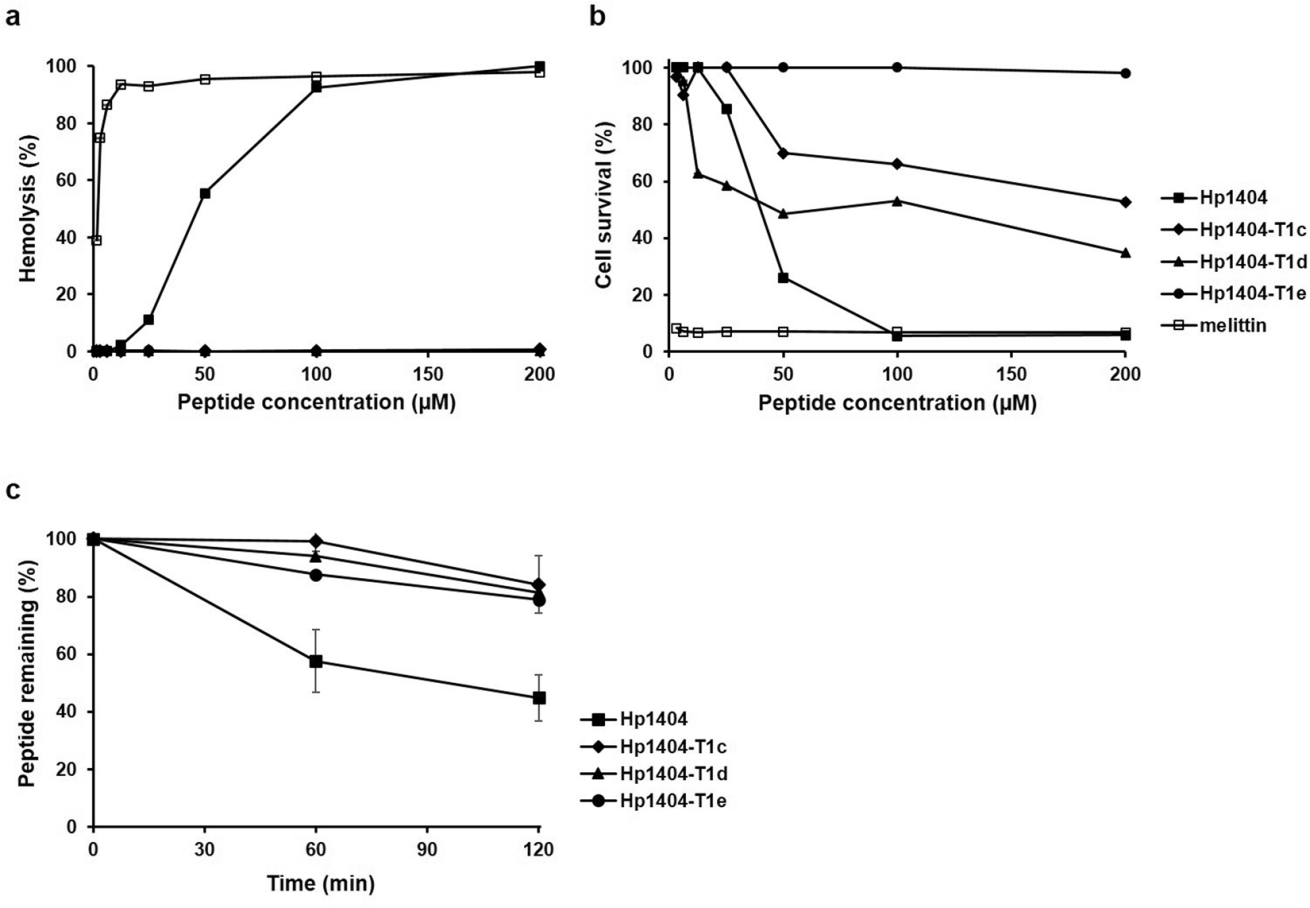
Toxicity and stability of synthetic peptides. (**a**) Hemolytic activity of peptides against mouse red blood cells. (**b**) Cytotoxicity of peptides was determined by examining their effect on the survival rate of HaCaT cells using the MTT assay. (**c**) Determination of the proteolytic stability of peptides plotted against time. Higher percentages indicate higher stability. The initial concentration for all four peptide samples was set to 100 µg/mL. Each experiment was repeated three times.

To investigate the toxicity of the peptides, their ability to lyse mouse red blood cells (RBCs) was examined (Fig. 2a). Melittin (bee venom)^24^, which was used as a positive control, caused complete hemolysis at 12.5 M. In the case of Hp1404, hemolysis rates were 2, 11, 55, 92, and 100%, at the respective concentrations of 12.5, 25, 50, 100, and 200 µM. In contrast, all analogue peptides did not induce substantial hemolysis, even at the higher tested concentration (200 µM).

Cytotoxicity of the peptides was assessed by investigating their toxic effects on HaCaT cells using the MTT method. Results are shown in Fig. 2b. Melittin, which was again used as a positive control, had a strong cytotoxic effect^25^; the survival rate was 11.29% at 1.56 µM, while no living cells were detected at 3.13 µM. Among the peptides, analogues displayed lower cytotoxicity compared to the parent peptide; the survival rates at 50 µM for Hp1404, Hp1404-T1c, Hp1404-T1d, and Hp1404-T1e were 30, 51, 33, and 100%, respectively. Based on the aforementioned results, all subsequent experiments were performed using Hp1404, Hp1404- T1c, Hp1404-T1d, and Hp1404-T1e.

### Bacterial killing kinetics

The effect of peptides on *P. aeruginosa* was investigated by studying time-kill kinetics (Fig. S3). In general, the peptides kill the bacteria at 4 hours while Hp1404-T1e kills 85% bacteria at 2 hours; Hp1404-T1e effectively killed all bacteria at 3 hours.

### Salt sensitivity

Salt environments have a negative impact on antimicrobial peptides. Therefore, the antibacterial activities of peptides were investigated following the addition of physiological concentrations of different salts for the sensitivity assay. Table 3 displays the MIC values obtained for each peptide tested in the presence of salt. Hp1404-T1e showed stable antibacterial activities, with MIC values ranging between 3.13 µM and 12.5 µM for most of the conditions tested. In comparison, Hp1404, Hp1404-T1c, and Hp1404-T1d displayed lower activities. The ability to resist to salt provides the Hp1404-T1e peptide with an advantage when it comes to selecting potential antimicrobial agents to be used in physiological environments.

**Table 3:** Antibacterial activities of synthetic peptides against *P. aeruginosa* 27853 under conditions mimicking physiological concentrations of salt.

Salt	Concentration	MIC (µM)			
		Hp1404	Hp1404-T1c	Hp1404-T1d	Hp1404-T1e
NaCl	50 mM	25	>25	12.5	6.25
	100 mM	>25	>25	12.5	6.25
	150 mM	>25	>25	25	12.5
CaCl_2_	1.25 mM	>25	>25	12.5	6.25
	2.5 mM	>25	>25	25	12.5
	5 mM	>25	>25	>25	>25
MgCl_2_	0.5 mM	25	12.5	3.13	3.13
	1 mM	25	12.5	6.25	3.13
	2 mM	>25	>25	12.5	12.5

### Trypsin stability

To investigate the protease stability of peptides, these were incubated with trypsin for 1 or 2 h. The products were separated using RP-HPLC. Supplementary Figure 5 displays the profiles obtained for Hp1404, Hp1404-T1c, Hp1404-T1d, and Hp1404-T1e, respectively, at all time points (0, 1, and 2 h). Stability comparisons were based on the percentages of remaining peptide determined by the HPLC peak area corresponding to the native peptide. The parent peptide is much more susceptible to proteolysis compared to its analogues, as the area of the peak corresponding to native Hp1404 is reduced faster. After 2 h of trypsin treatment, only 50% of Hp1404 remains. In contrast, more than 80% of the analogue peptides had escaped proteolytic cleavage at the same time point (Fig. 2c).

### Inhibition and visualization of biofilms

To investigate the antibiofilm activity of the peptides, biofilm formation was examined in the presence of each peptide. *P. aeruginosa* ATCC 27853 selected to perform the biofilm formation related experiments^26^, Hp1404, Hp1404-T1c, Hp1404-T1d, and Hp1404-T1e inhibited biofilm formation at concentrations of 25, 25, 12.5, and 12.5 µM, respectively. We also examined the antibiofilm activity of the peptides against multidrug-resistant *Pseudomonas aeruginosa* strains. Minimal biofilm inhibitory concentration (MBIC) values ranged from 6.25 to >50 µM; Hp1404-T1e had the strongest antibiofilm activity among the tested peptides (Table 4).

**Table 4:** MBIC values of the parent Hp1404 peptide and three of its analogues, as well as ciprofloxacin, against *P. aeruginosa* strains.

Peptide	MBIC (µM)				
	Hp1404	Hp1404-T1c	Hp1404-T1d	Hp1404-T1e	Ciprofloxacin
*P. aeruginosa* 27853	25	25	12.5	12.5	—
*P. aeruginosa* 434	25	50	12.5	12.5	>400
*P. aeruginosa* 559	50	25	12.5	12.5	>400
*P. aeruginosa* 778	25	25	12.5	6.25	>400
*P. aeruginosa* 1034	25	25	12.5	6.25	>400
*P. aeruginosa* 1162	25	12.5	6.25	6.25	>400
*P. aeruginosa* 3543	6.25	>50	>50	50	>400
*P. aeruginosa* 3399	12.5	25	25	12.5	>400
*P. aeruginosa* 4007	25	>50	50	12.5	>400

**Figure 3:**
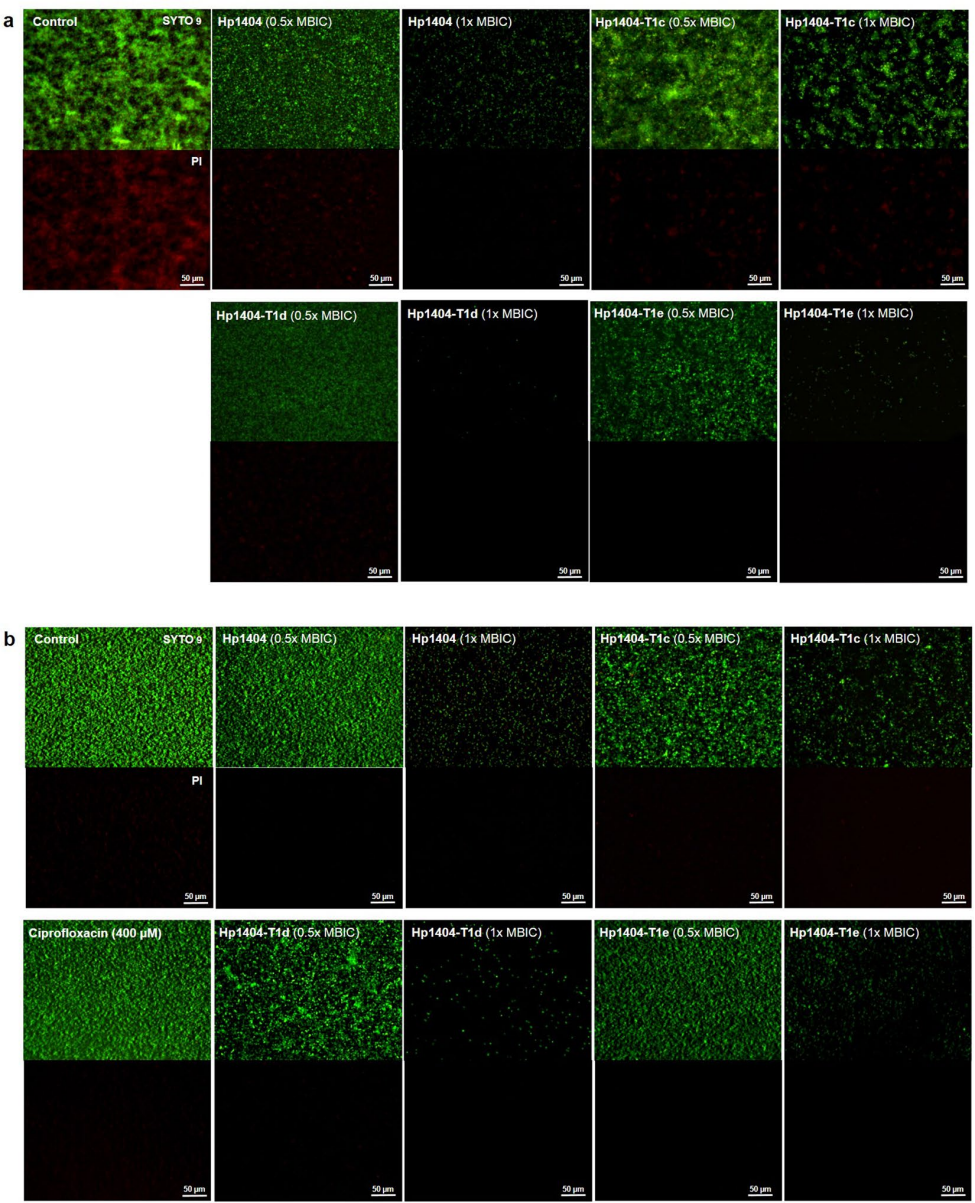
Fluorescence microscopy images of live/dead stained *P. aeruginosa* biofilms after treatment with peptides at 0.5× or 1× MBIC. Living cells are stained in green (SYTO9 dye), while dead cells are stained in red (propidium iodide). (**a**) *P. aeruginosa* ATCC 27853 (**b**) *P. aeruginosa* 1034. Scale bar = 50 µM.

Finally, we used LIVE/DEAD BacLight staining of bacteria to visualize and examine the viability of cells in biofilms of both standard (ATCC 27853) and multidrug-resistant (1034) strains of *P. aeruginosa* after treatment with peptides at 0.5× and 1× MBIC. At both concentrations, the peptides significantly impacted biofilm formation, although the lowest concentration was unable to completely remove the biofilm. As expected, ciprofloxacin did not inhibit biofilm formation by the MRPA strain, even at 400 µM (Fig. 3).

### LPS binding assay

**Figure 4:**
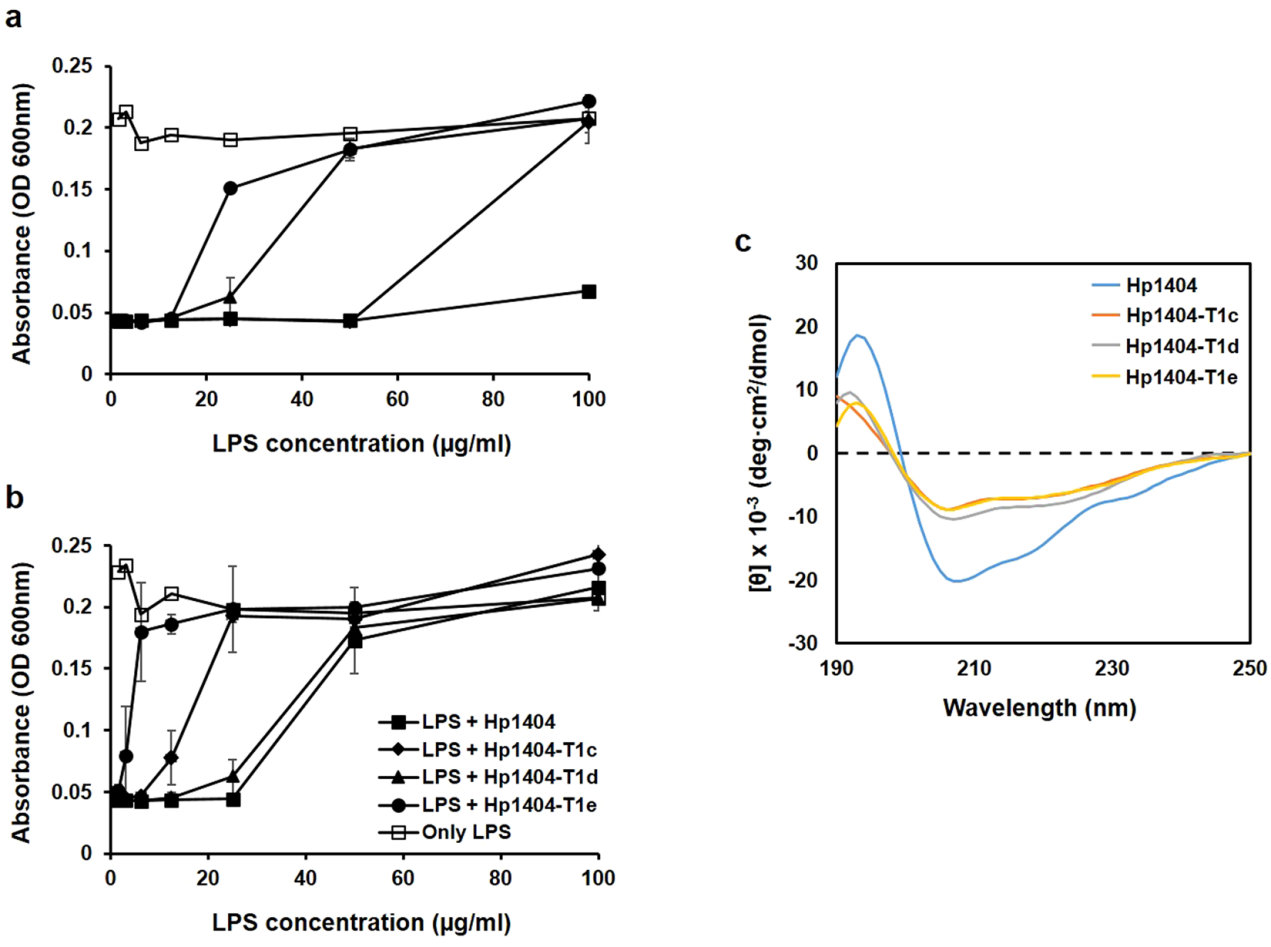
LPS binding assay. Affinities of peptides toward LPS were examined by determining the effect of increasing LPS concentrations on their antimicrobial activities at (**a**) 1× MIC, and (**b**) 2× MIC, against *P. aeruginosa*. (**c**) CD spectra of peptides in 0.1% LPS suspensions. The peptide concentration was fixed at 40 µM. Each experiment was repeated three times.

To investigate whether lipopolysaccharides (LPS) bind to peptides, we examined their antimicrobial activities at 1× and 2× MIC against *P. aeruginosa*, in the presence of increasing LPS concentrations. As expected, LPS alone do not affect the proliferation of *P. aeruginosa*. In the case of Hp104, the absorbance at 600 nm remained the same, irrespective of LPS concentration, indicating that LPS do not affect the ability of the parent peptide to suppress bacterial growth. In contrast, there is a clear negative effect of LPS on the antimicrobial action of the peptides, beginning at LPS concentrations of 100 µg/mL (Hp1404-T1c), 50 µg/mL (Hp1404-T1d), and 25 µg/mL (Hp1404-T1e). The effect becomes more pronounced as the LPS concentration increases further (Fig. 4a). Antibacterial activities of Hp1404, Hp1404-T1c, Hp1404-T1d, and Hp1404-T1e were significantly lower after the addition of LPS at concentrations of 50, 25, 50, and 6.25 µg/mL, respectively (Fig. 4b).

We used CD spectroscopy to investigate the secondary structures of the parent peptide and its analogues in the presence of LPS from *P. aeruginosa*. As shown in Fig. 4c, the spectra of all peptides displayed a positive peak at 192 nm, and two negative peaks (at 208–210 nm and 220–225 nm). This pattern indicates the adoption of a helical conformation, especially in the case of Hp1404. Our results suggest that the peptides interact with LPS to produce an α-helix structure.

### Secondary structures of Hp1404 and its analogues

**Figure 5:**
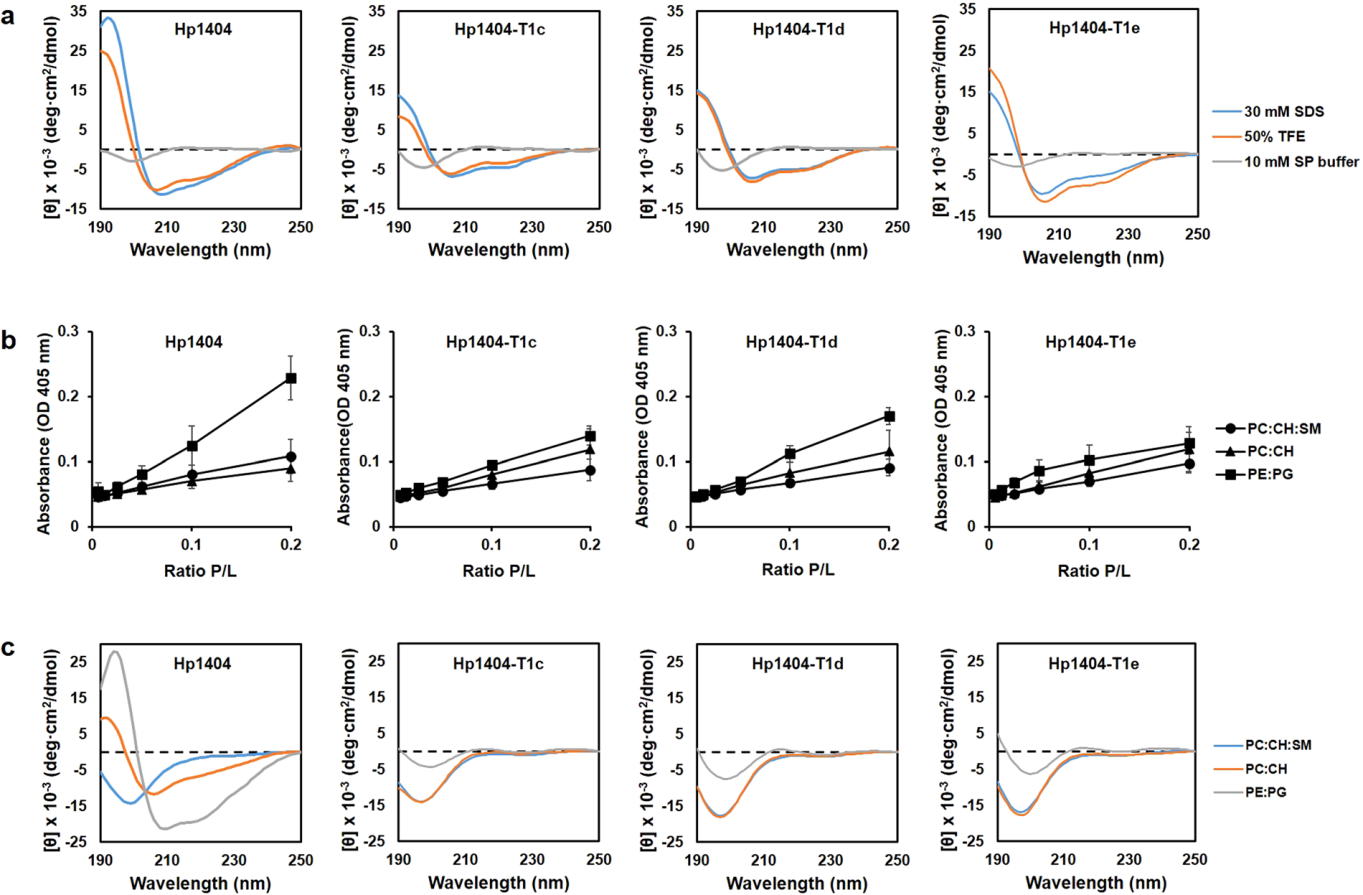
Circular dichroism (CD) spectra and large unilamellar vesicle (LUV) aggregation. (**a**) Peptide levels were measured in 30 mM SDS, which mimics the negatively charged prokaryotic membrane environment, or 50% TFE, which mimics the hydrophobic environment of the microbial membrane. (**b**) LUV aggregation. Solutions containing various concentrations of peptides were added to 400 µM suspensions of PE:PG (7:3, w/w), PC:CH:SM (1:1:1, w/w), or PC:CH (2:1, w/w) LUVs. (**c**) CD spectra of peptides in the presence of LUVs composed of PC:CH:SM (1:1:1, w/w), PC:CH (10:1, w/w), or PE:PG (7:3, w/w) mixtures.

To determine the secondary structure of peptides in membrane-mimicking environments, circular dichroism (CD) spectra were obtained from peptides dissolved at a final concentration of 40 µM in the following solutions: 10 mM sodium phosphate buffer, pH 7.2 (mimics the aqueous environment); 30 mM sodium dodecyl sulfate (SDS, mimics the negatively charged prokaryotic membrane environment), and 50% trifluoroethanol (TFE, mimics the hydrophobic environment of the microbial membranes). The spectra are shown in Fig. 5a. In the sodium phosphate solution, the parent peptide and its analogues displayed a positive peak at 212 nm and a negative peak at 195 nm, indicating a random coil conformation. In the other two solutions, all peptides displayed spectra corresponding to α-helices, with two negative peaks at about 208 nm and 222 nm, and a positive peak at 190 nm, suggesting that the peptides adopted an α-helical conformation in the membrane-mimicking environments.

### CD spectra analyses and liposome aggregation

We also investigated the peptide structure in the presence of large unilamellar vesicles (LUVs) composed of PE:PG (phosphatidylethanolamine:polyethylene glycol) to mimic bacterial membranes, PC:CH:SM (phosphatidylcholine:cholesterol:sphingomyelin) to mimic mammalian cell membranes, or PC:CH (phosphatidylcholine:cholesterol) to mimic erythrocyte membranes (Fig. 5b). Hp1404 adopted α-helical structures in the presence of PE:PG and PC:CH liposomes, whereas it displayed a random coil conformation when mixed with PC:CH:SM liposomes. In contrast, all three analogues tested (Hp1404-T1c, Hp1404-T1d, and Hp1404-T1e) exhibited random coil conformations in the presence of all liposome types (Fig. 5c).

Finally, we assessed the ability of the four peptides to induce liposome aggregation. Peptides were mixed with liposome suspensions (prepared from PE:PG, PC:CH, or PC:CH:SM mixtures) at various peptide/liposome (P/L) ratios (Fig. 5b). In all cases, higher P/L ratios led to larger increases in turbidity. PC:CH and PC:CH:SM LUVs were much more resistant to aggregation caused by any of the four peptides compared to PE:PG liposomes. Moreover, Hp1404 was much more effective in promoting aggregation of PE:PG liposomes compared to its analogues. At the higher tested P/L ratio (0.2), the parent peptide increased the turbidity by almost 25%, whereas the respective percentages for the other three peptides were well below 20%.

### Calcein leakage assay

**Figure 6:**
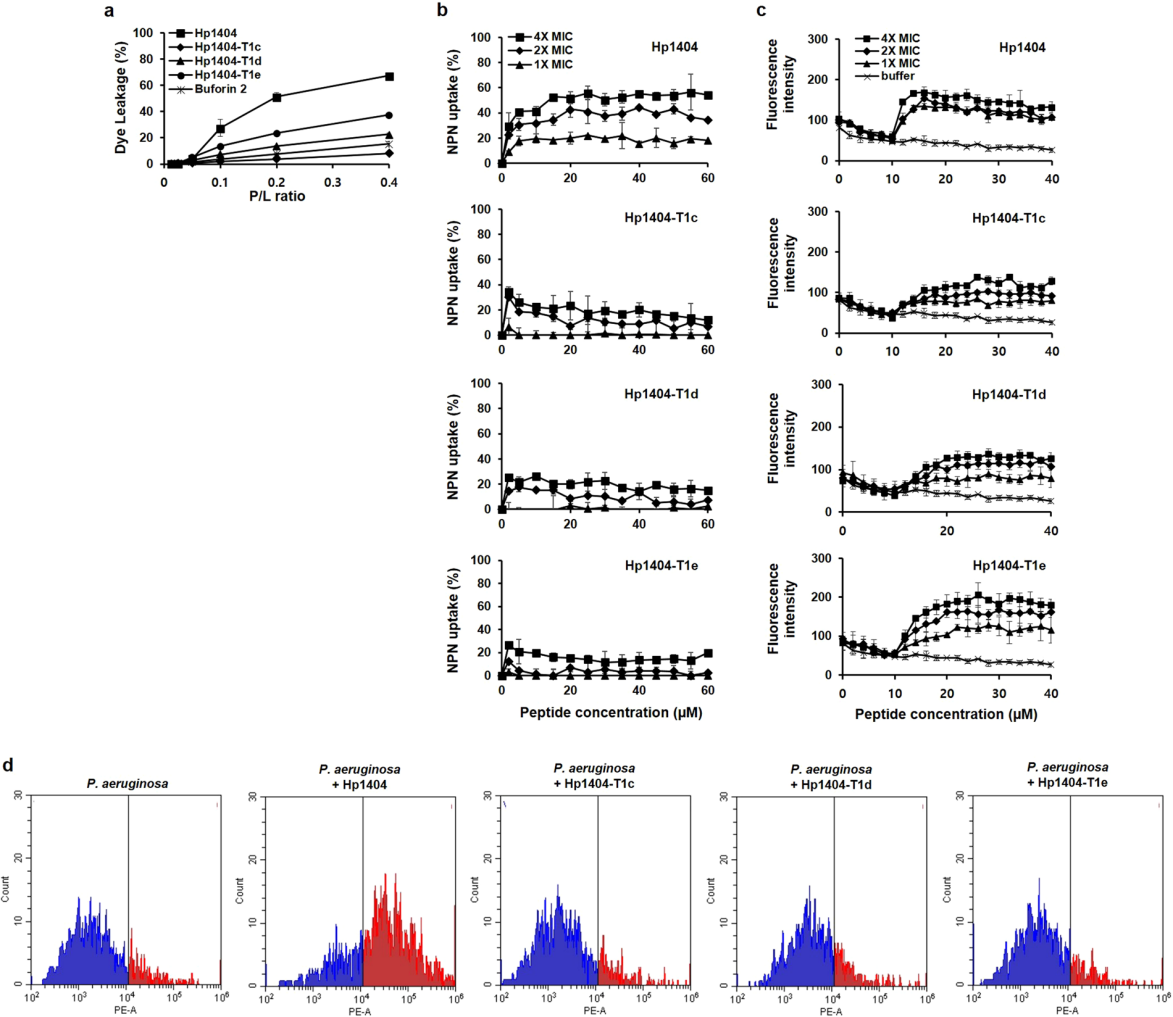
(**a**) Calcein leakage from PC:PG LUVs (9:1, w/w, negatively charged) in the presence of the synthetic peptides. (**b**) The outer membrane permeability of the peptides Hp1404, Hp1404-T1c, Hp1404-T1d, and Hp1404-T1e, as determined using the fluorescent dye (NPN) assay. (**c**) The cytoplasmic membrane depolarization, as measured by release of DisC3-5 dye. (**d**) Peptide-induced bacterial membrane permeabilization evaluated through FACS analysis on cells stained with PI (10 µg/mL) at 4 °C for 2 h.

The leakage assay is an experiment carried out to assess pore formation and membrane perturbation. We monitored the release of calcein from negatively charged PC:PG (9:1) LUVs, which mimic a bacterial membrane. At a peptide concentration of 4 µM, P/L ratio of 0.4, Hp1404 induced a release of about 70% of the calcein, while the calcein release provoked by the analogue peptides stayed below 30% (Fig. 6a). Therefore, Hp1404 exhibited significantly higher leakage activity than the analogue peptides.

### Outer membrane permeabilization activity of peptides against *P. aeruginosa*

We investigated the ability of the peptides to disrupt bacterial membranes using the NPN uptake assay. The hydrophobic probe NPN is used to assess the permeabilization of the outer membrane of gram-negative bacteria. NPN exhibits weak fluorescence in aqueous environments, but produces a strong signal in hydrophobic environments such as the lipid layer of the outer bacterial membrane, to which it gains access when the membrane is disrupted. Incubation with 4× MIC Hp1404 for 5 min increased NPN uptake by 40%, indicating that the parent peptide was able to effectively permeabilize the membrane. The increases in NPN fluorescence induced by the analogue peptides were significantly lower (Fig. 6b) than the one caused by Hp1404, suggesting that the analogues had a lower outer membrane permeabilizing activity compared to the parent peptide.

### Depolarization of cytoplasmic membrane in *P. aeruginosa*

The membrane potential-sensitive dye DisC_3_-5 was used to evaluate the ability of the peptides to depolarize the bacterial cytoplasmic membrane (Fig. 6c). DisC_3_-5 accumulates in polarized cytoplasmic membranes, whereas disruption of the membrane potential releases the probe, resulting in an increase in fluorescence intensity. After a 10-min stabilization period, peptides were added and changes in membrane potential were monitored for another 30 min. All peptides dose-dependently increased fluorescence intensity, suggesting that they had a significant membrane depolarization effect on *P. aeruginosa*. Hp1404 or Hp1404-T1e at 4× MIC increased fluorescence intensity to almost 200, whereas 4× MIC Hp1404-T1c and Hp1404-T1d increased fluorescence intensity up to 150.

### PI uptake assay

Propidium iodide (PI) uptake was performed to observe the permeabilization activity on the *Pseudomonas aeruginosa* membrane. Increasing PI fluorescence indicates the degree of permeability of the bacterial membrane (Fig. S6). Melittin, which is known to quickly disrupt the membrane, generated an increased in PI fluorescence within 2 min. In contrast, the fluorescence generated by ciprofloxacin was low, indicative of the absence of effect on membrane permeabilization. The permeabilization effect of peptides was concentration-dependent. Similarly to melittin, Hp1404 exhibited an activity on the membrane, while in contrast, analogue peptides at 1× MIC showed relatively low activities.

### FACS analysis

FACS analysis of propidium iodide-stained bacteria was applied to compare the effect of Hp1404 and one of its analogues, Hp1404-T1e, on the integrity of bacterial membranes, as described by Jang *et al*.^27^. Propidium iodide (PI) is a membrane impermeant dye that binds to double-stranded DNA by intercalating between base pairs. In the absence of peptides, the percentage of PI-positive *P. aeruginosa* cells was extremely low (15.75%), indicating intact cell membranes. Treatment with 2× MIC Hp1404 resulted in a dramatically higher percentage for *P. aeruginosa* (71.77%) (Fig. 6d). Treatment with Hp1404-T1c, Hp1404-T1d, and Hp1404-T1e increased the percentages of PI-positive *P. aeruginosa* cells (16.17, 21.10, and 16.22%, respectively), but to a much lower extent. These results suggest that both Hp1404 and Hp1404-T1e damage bacterial membranes; however, the parent peptide is much more efficient in doing so.

### DNA binding assay

After revealing the significant differences among the peptides with respect to their effects on bacterial membranes, we investigated their intracellular effects; specifically, we evaluated their DNA binding ability using the gel retardation assay, as previously described^28^.

**Figure 7:**
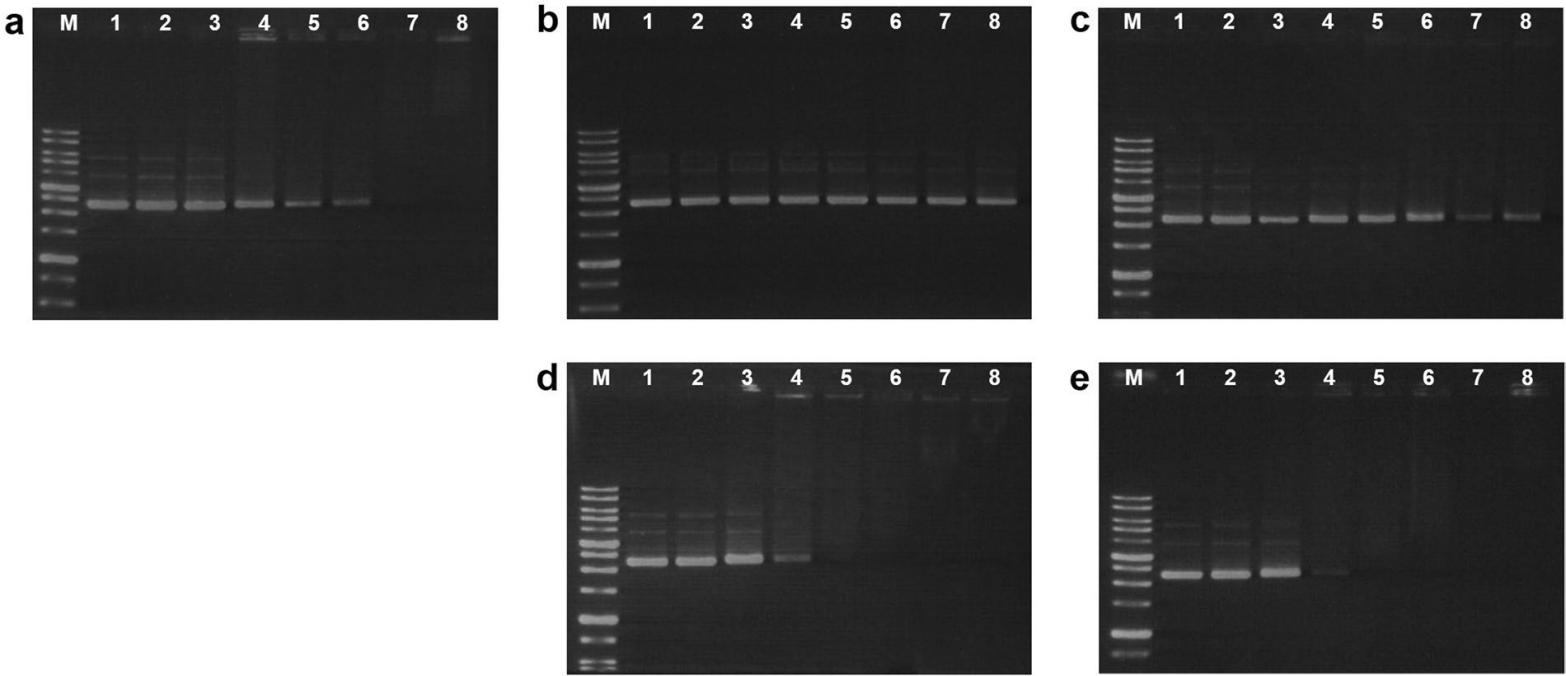
DNA binding assay. Peptide-DNA binding was assayed by evaluating the peptide-induced inhibition of plasmid DNA migration. (**a**) Buforin 2, (**b**) Hp1404, (**c**) Hp1404-T1c, (**d**) Hp1404-T1d, or (**e**) Hp1404-T1e were incubated with 260 ng pRSETB at 37 °C for 10 min, followed by electrophoresis in a 1% agarose gel at 100 V. The peptide:DNA ratio in each lane was as follows: lane 1, only DNA; lane 2, 0.25:1; lane 3, 0.5:1; lane 4, 1:1; lane 5, 1.5:1; lane 6, 2:1; lane 7, 3:1; lane 8, 4:1.

Buforin 2, used as a positive control, is known to bind to DNA and kill bacteria without damaging the cell wall^29^. As seen in Figs 7 and S7, Hp1404 and Hp1404-T1c did not bind to DNA. In contrast, Hp1404-T1c and Hp1404-T1e exhibited a clear inhibitory effect on DNA migration at peptide:DNA ratios of 1.0 and higher. These results suggest that Hp1404-T1c and Hp1404-T1e can interact with DNA, similarly to buforin 2 (Figs 7a and S7a)^30^.

### Efficacy of Hp1404-T1e ***in vivo***

**Figure 8:**
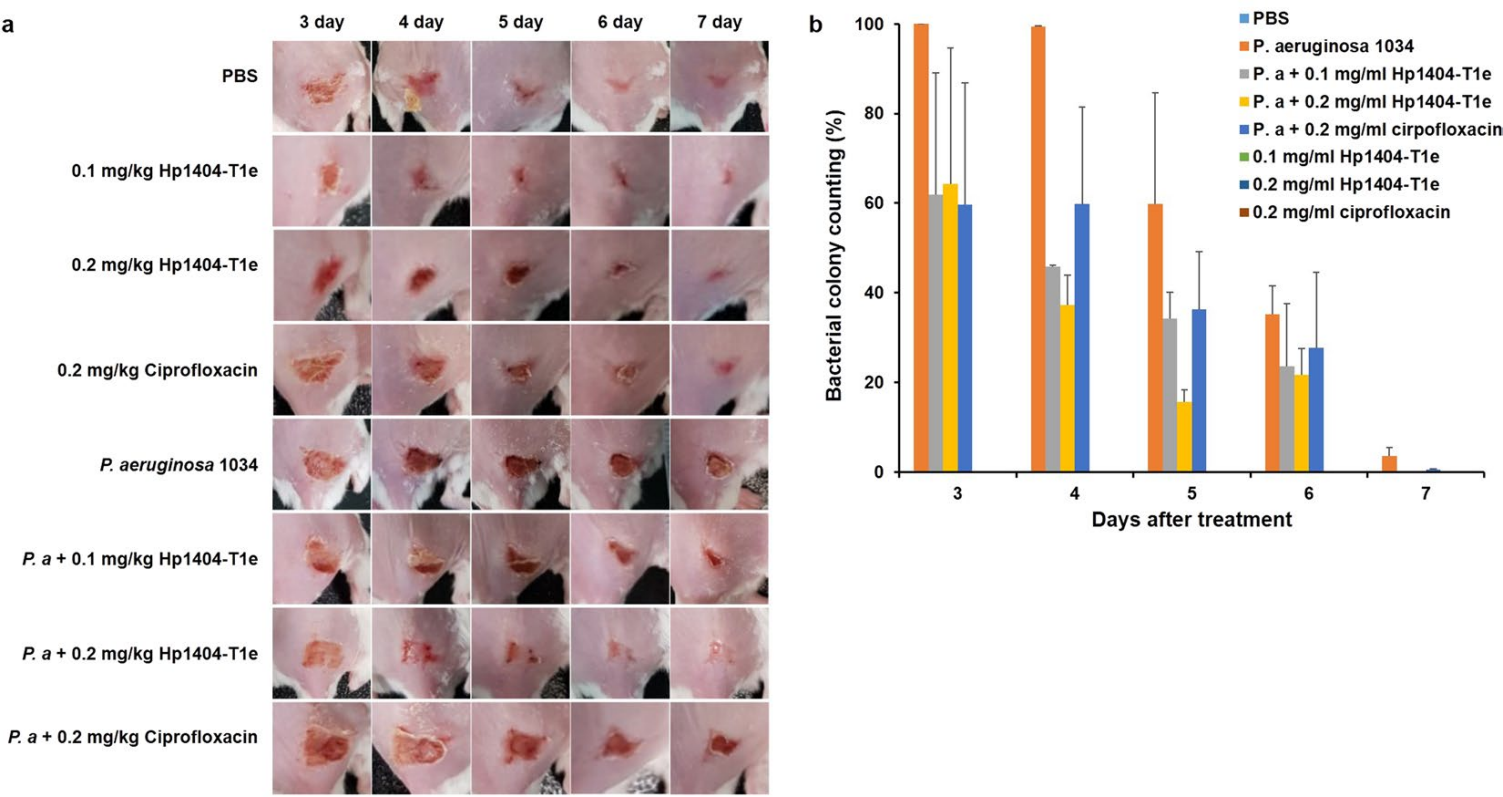
Antibacterial effect of Hp1404-T1e *in vivo*. (**a**) Image of the infected skin after 3, 4, 5, 6, or 7 days. (**b**) Percentage of bacterial colonies in *P. aeruginosa* infected skin. Hp1404-T1e was used at a concentration of 0.1 mg/ml and 0.2 mg/ml after *P. ae ruginosa* 1034 infection.

In order to observe the antimicrobial effect of Hp1404-T1e *in vivo*, BALB/c mice were used (Fig. 8). In the absence of bacterial infection, PBS treated mice, Hp1404-T1e treated mice and ciprofloxacin-treated mice served as controls. Compared with the control group, *P. aeruginosa*-infected mice showed colonies until 4 days. The number of colonies in Hp1404-T1e treated mice decreased to 60% after 3 days and to 40% after 4 days, but ciprofloxacin showed a decrease of about 60% until 4 days. On the 5^th^ and 6^th^ days, the number of bacterial colonies was slightly reduced in *P. aeruginosa*-infected mice and ciprofloxacin-treated mice, but Hp1404-T1e treated mice bacterial colony formation was strongly inhibited. On day 7, mice treated with Hp1404-T1e, the bacteria were completely killed, whereas *P. a*-infected mice and ciprofloxacin treated mice did not kill the bacteria.

## Discussion

The emergence and rapid worldwide spread of antibiotic-resistant bacteria poses major public health issues^31^. New antibacterial agents are being developed to counter the growing resistance of bacteria against conventional antibiotics. AMPs, which are essential elements of the human innate immune system, are excellent candidates for the development of novel therapeutic agents.

AMPs are found in a variety of organisms (e.g., insects, fungi, plants, and mammals), and characterized by cationic amphipathic properties. Up to now, 50 peptides have been isolated from the scorpion venom and most of them exhibit broad spectrum antimicrobial activities against bacteria, including multidrug-resistant strains^32^. However, scorpion peptides are toxic toward eukaryotic cells and therefore, it is necessary to develop AMPs that maintain high activity while displaying lower toxicity^33,34^.

In a previous study, Hp1404, composed of 14 amino acids and displaying an amphipathic and α- helical structure, was isolated from scorpion. However, although this peptide displays a potent antimicrobial activity against gram-positive bacteria, including *Staphylococcus aureus*, it also exhibits toxicity toward eukaryotic cells^20^.

In this study, we designed new analogue peptides (Hp1404-T1, Hp1404-T1a, Hp1404-T1b, Hp1404-T1c, Hp1404-T1d, and Hp1404-T1e) based on Hp1404. These peptides are composed of 12 amino acids after removal of Glycine 1 and Phenylalanine 14 from the parent peptide. Subsequently, the analogue peptides form a pronounced amphipathic structure, displaying higher cationic properties, as a result from the substitution of the removed residues with lysine and leucine residues^35^. Peptides comprising the hydrophilic amino acid leucine and positively charged lysine residues have strong antimicrobial activity and form a helix structure^36,37^. The hydrophobic amino acid Leucine increases the hydrophobicity in an amphipathic structure, thereby conferring an important role in antimicrobial activity through the interaction with bacterial membranes^38^. The lysine residue, a positively charge amino acid, enhances the binding between AMP and the negatively charged bacterial membrane by increasing the electrostatic attraction^39^. Therefore, the leucine and lysine residues were located on the hydrophobic and hydrophilic faces, respectively (Fig. 1). In the 3D representation, the N-terminal random coil structure disappears and a strong α-helical structure is formed. As a result, the analogue peptides display a well-defined amphipathic α-helical structure, with higher hydrophobic moment, and increased net positive charge^37,40^.

We examined the antibacterial activities of Hp1404 and its analogues against microorganisms. The parent peptide and three of the analogues (Hp1404-T1c, Hp1404-T1d, and Hp1404-T1e) exhibited antimicrobial activities. The parent peptide displayed antibacterial activity against gram-positive bacteria, including methicillin-resistant *staphylococcus aureus* (MRSA); however, it exhibited hemolytic and toxic activities toward mammalian cells. The antimicrobial activity assays demonstrated that Hp1404-T1e was the analogue with the most potent activity against *Pseudomonas aeruginosa* strains. In contrast, the conventional antibiotic, ciprofloxacin, showed no antibacterial activity, even at high concentrations (Table 2). At active concentrations, Hp1404-T1e displayed no hemolytic activity and no toxicity toward mouse RBCs or HaCaT cells, respectively. We also showed that Hp1404-T1e was not toxic through calcein leakage assay, indicating that Hp1404-T1e could be developed into a safe therapeutic agent (Fig. S4).

The development of antimicrobial peptides involves overcoming several limiting factors. Firstly, antimicrobial activity decreases in high-salt conditions, such as those found in physiological environments. Secondly, their sensitivity toward digestion by proteases can also represent a major drawback^41^. After testing their stability upon trypsin treatment for 2 h, the analogue peptides proved to be more resistant toward proteolytic degradation than Hp1404 (Fig. S5), suggesting that Hp1404-T1e might be able to overcome this common limitation^42^. Additionally, we confirmed that the activity of this analogue peptide was not overly impacted by high-salt environments, which is an important feature regarding its use under physiological conditions (Table 3)^43^. Taken together, these results show that Hp1404-T1e is stable *in vivo* and can be used as a therapeutic agent.

Bacterial biofilms are involved in a wide range of microbial infection events. A typical biofilm infection is usually chronic because of the low sensitivity of constituting cells toward antibiotics^44^. *P. aeruginosa* is known as a major biofilm-forming pathogen. We investigated the ability of the peptides to inhibit *P. aeruginosa* biofilm formation (Table 4). Hp1404-T1e could inhibit biofilm formation at a concentration lower than the one required when using the conventional antibiotic. The degree of biofilm inhibition was visualized using SYTO9/PI staining. As a result, we conclude that the peptide analogues are effective agents against *P. aeruginosa* biofilm-related infections (e.g., cystic fibrosis, keratitis)^45^.

LPS, also known as endotoxins, are located on the outer membrane of gram-negative bacteria and can cause inflammations^46^. Peptide-LPS interaction is important toward allowing bacterial death and reducing inflammation^47^. Owing to their cationic properties (positive net charge), AMPs can bind to, and accumulate on, the surface of bacteria that contain anionic polymers such as LPS. The CD spectra obtained suggest that Hp1404 and its analogues display an α-helical structure when bound to LPS. Unlike Hp1404, which antimicrobial activity was not affected by LPS, the analogue peptides showed lower activities in the presence of 100 µg/mL LPS, suggesting that LPS binds to the peptides and interfere with their antibacterial activity. The absorbance recorded after adding 2× MIC of peptides increased compared to the value obtained at 1× MIC. We conclude that increasing the net charge significantly enhances peptide affinity toward the bacterial surface. Taken together, these data suggest that peptides could prove effective against LPS-induced inflammations.

Antimicrobial peptides are reported to act by forming α-helical, β-sheet, and random coil structures^48,49^. Even though peptides were predicted to form α-helices (Fig. 1b), the secondary structure is dependent on the environment. Thus, we used CD spectroscopy to investigate the peptide conformation in different solutions. Peptides formed random coils in aqueous solutions and α-helixes in bacterial membrane-mimicking environments. Furthermore, LUVs were used to investigate the relationship between membrane lipid composition and AMP-induced aggregation. CD spectroscopy was used to investigate the peptide secondary structure in the presence of LUVs. These data suggest that a high concentration of Hp1404 is required to allow the α-helical structure to appear in the presence of PE:PG (bacterial membrane mimicking) and PC:CH (mimicking RBCs) liposomes. There is no relationship with the 3D structure (Fig. 1b). Hp1404 showed strong affinity to PE:PG liposomes compared to analogue peptides. In contrast, Hp1404-T1c, Hp1404-T1d, and Hp1404-T1e only displayed weak affinities for these LUVs. In the cases of PC:CH and PC:CH:SM liposomes, neither of the four tested peptides caused aggregation. These data indicate that low concentrations of the peptides do not cause toxicity. Combined with the results obtained from the CD spectroscopy assay, we conclude that Hp1404 acts strongly on the bacterial membrane while the effects induced by the analogue peptides are very limited.

In a more detailed mechanism study, three Hp1404 analogues were compared to the parent peptide with respect to their biological activities and action mechanisms. To explore the action mechanism of peptides, their effects on bacterial membranes were investigated using the calcein leakage assay, the NPN outer membrane permeabilization assay, the DisC_3_-5 cytoplasmic membrane depolarization assay, and PI FACS analysis. PC:PG liposomes were used to mimic negatively charged bacterial membranes. The analogue peptides did not induce calcein leakage from these LUVs, whereas Hp1404 did. Hp1404 significantly increased NPN fluorescence, implying its damaging effect on the outer membrane. The analogue peptides caused a much smaller increase in NPN uptake percentage compared to the parent peptide, indicating the absence of effect on outer membrane permeabilization. In *P. aeruginosa*, incubation with parent or analogue peptides resulted in similar levels of fluorescence intensity being generated. Taken together, these results suggest that there is a difference between the parent peptide and its analogues with respect to their effect on membranes. FACS analysis showed that Hp1404 damaged membranes while Hp1404-T1e did not. Based on these results, we investigated the potential presence of intracellular peptide targets using the DNA binding assay^50^. Surprisingly, Hp1404-T1d and Hp1404-T1e interacted with DNA, suggesting that their differences with the parent peptide may imply the existence of a distinct action mechanism^51,52^.

We observed the antibacterial effect of Hp1404-T1e *in vivo* and found that Hp1404-T1e has an inhibitory activity against *P. aeruginosa in vivo* compared to ciprofloxacin antibiotics; thus, Hp1404-T1e showed potential as a good therapeutic agent.

In conclusion, among the analogue peptides, Hp1404-T1e exhibited potent activity against *P. aeruginosa* strains. The peptides displayed lower hemolytic and toxic activities towards mammalian cells compared to the parent peptide. Hp1404-T1e showed better antibiofilm formation properties than the parent peptide. Moreover, Hp1404 kills bacteria through membrane disruption, while Hp1404-T1e may be able to enter the cells and interact with their DNA. Furthermore, Hp1404-T1e has high stability against salts and enzymes and has antibacterial activity *in vivo*. Overall, Hp1404-T1e is a promising candidate for the development of a novel therapeutic agent against *P. aeruginosa* infections.

## Materials and Methods

### Materials

Dimethyl sulfoxide (DMSO), 3-(4,5-dimethylthiazole-2-yl)-2,5-diphenyltetrazolium bromide (MTT), lipopolysaccharide (LPS) from *P. aeruginosa*, *N*-phenyl-1-naphthylamine (NPN), 3,3′-dipropylthiadicarbocyanine iodide (DiSC_3_-5), propidium iodide (PI), and trypsin/ciprofloxacin were obtained from Sigma-Aldrich (St Louis, MO, USA).

Gram-negative (*Pseudomonas aeruginosa* ATCC 27853) bacterial strains were obtained from the ATCC (American Type Culture Collection; Manassas, VA, USA). Multidrug-resistant strains (*Pseudomonas aeruginosa* 138, 431, 434, 557, 559, 778, 1034, 1162, 3290, 3399, 3592, 3904, 4007, 4319, 4891, 5018, and 671973) were obtained from the Chonnam National University Hospital (Gwangju, South Korea).

### Peptide design and sequence analysis

The peptides were designed by truncating and substituting residues, based on the helical wheel diagram and three-dimensional structure of Hp1404. Projections of the predicted three-dimensional structures were constructed online using the Mobyle@RPBS bioinformatics portal (http://mobyle.rpbs.univ-paris-diderot.fr/cgi-bin/portal.py#welcome), whereas the HeliQuest site (http://heliquest.ipmc.cnrs.fr) was used to create helical wheel diagrams and determine the relative hydrophobic moments of the peptides.

### Antimicrobial activity assay

We examined the activity of each peptide against both *pseudomonas aeruginosa* and multidrug-resistant strains. The minimal inhibitory concentrations (MICs) of peptides were determined using the broth micro-dilution assay as described previously^53^. Briefly, bacterial strains were cultivated with shaking overnight at 37 °C. The next day, they were diluted to a density of 2 × 10^5^ CFU/mL with growth medium. Peptides were serially diluted with 10 mM sodium phosphate buffer. 50 µL bacterial aliquots were mixed with an equal volume of peptide solution, and incubated at 37 °C for 18–24 h. Inhibition of bacterial growth was determined by measuring the absorbance at 600 nm using a Versa Max microplate reader (Molecular Devices, Sunnyvale, CA, USA). The test was performed in triplicate.

### Hemolysis and cytotoxicity assay

The hemolytic activities of the peptides were determined as previously described^54^. Fresh mouse red blood cells (RBCs) were washed and diluted to 8% with PBS. RBC suspension was added to 96-well plates (100 µL/well), mixed with an equal volume of serially diluted peptides, and incubated at 37 °C for 1 h with gentle shaking. The plate was centrifuged and the supernatant was transferred to a 96-well plate. The absorbance at 414 nm was measured with a Versa-Max ELISA reader (Molecular Devices). PBS and 0.1% Triton X-100 were used as negative and positive controls, respectively. The experiment was performed in triplicate. The hemolysis rate was calculated using the following formula^55^: % Hemolysis = [(Abs 414 nm in the peptide solution-Abs 414 nm in PBS)/(Abs 414 nm in 0.1% Triton X-100-Abs 414 nm in PBS)] × 100

We used the MTT assay to evaluate the toxicity of the peptides toward human skin epithelial cells (HaCaT) as previously described^56^. Briefly, HaCaT cells were seeded at a density of 2 × 10^4^ cells per well in 96-well microtiter plates in Dulbecco’s modified Eagle’s medium (DMEM) containing 10% foetal bovine serum (FBS) at 37 °C under 5% CO2 atmosphere. The next day, peptides were added to each well at various final concentrations (higher concentration of 200 µM). After 24 h incubation, culture supernatants were removed. 0.5 mg/mL MTT was added to each well, and the plates were incubated for 4 h at 37 °C. The medium was replaced by dimethyl sulfoxide (DMSO) to dissolve the formazan crystals formed in the wells. The absorbance at 570 nm was measured using a Versa-Max microplate ELISA reader (Molecular Devices). The viability rate was calculated using the following formula: Cell viability (%) = (absorbance of treated sample)/(absorbance of control) × 100.

### Salt sensitivity

The effects of various cations on the antibacterial activities of the parental and analogue peptides against *P. aeruginosa* 27853 were evaluated as previously described^57^. Bacteria was treated with peptides at a final concentration 25 µM in the presence of physiological salts such as NaCl (50 mM, 100 mM, and 150 mM), CaCl_2_ (1.25 mM, 2.5 mM, and 5 mM), or MgCl_2_ (0.5 mM, 1 mM, and 2 mM). The MIC values were determined as described above.

### Trypsin stability assay

Each sample with trypsin was analysed by RT-HPLC on a Jupiter C18 column (4.6 × 250 mm, 300 Å, 5 µm; Phenomenex). Stability was expressed as the percentage of remaining peptide, which in turn was determined by comparing the HPLC peak areas, corresponding to the peak detected for the native peptide, before and after trypsin treatment.

### Antibiofilm activity assay and SYTO9/PI staining

To evaluate the ability of bacterial strains to form biofilms either in the absence or presence of peptides, antibiotic-susceptible *P. aeruginosa* (ATCC 27853), and various strains of MDR *P. aeruginosa* were cultured in nutrient broth (NB). Bacterial suspensions (5 × 10^5^ CFU/mL) with 0.2% glucose were added to 96-well tissue culture plates, mixed with peptide solution (at a final concentration of 25 µM), and incubated for 24 h at 37 °C. After the incubation, culture supernatants were discarded and wells were gently washed with PBS to remove planktonic cells. The biofilms were fixed with 100% methanol for 15 min, stained with 0.1% crystal violet (CV) for 1 h, and rinsed three times with distilled water (dH_2_O). After the addition of 95% ethanol, biofilm mass was quantified by measuring OD at 595 nm using a Versa-Max microplate ELISA reader (Molecular Devices). Results were expressed as the percentage of biofilm formed with respect to the control. We also calculated the minimum biofilm inhibitory concentration (MBIC), which was defined as the lowest peptide concentration at which biofilm formation was completely inhibited. All experiments were performed at least in triplicate.

Visualization of the remaining bacteria in the biofilm was achieved using the LIVE/DEAD BacLight Bacterial Viability Kit (Molecular Probes, Eugene, OR, USA), and an IX71 inverted fluorescence microscope (Olympus, Tokyo, Japan) was used for observation^58,59^. Briefly, bacterial suspensions (5 × 10^5^ CFU/mL) were incubated with 0.5× MBIC or 1× MBIC of the peptides, for 24 h at 37 °C. Following incubation, biofilms were stained with pre-mixed dye (SYTO-9 and propidium iodide) for 30 min in the dark; images were obtained using the aforementioned microscope.

### LPS binding assay

To study the effect generated by interactions between lipopolysaccharides (LPS) and peptides, we monitored the antimicrobial activity against *P. aeruginosa* ATCC 27853. The LPS (final concentration 100 µg/mL) solution was serially diluted in 10 mM sodium phosphate buffer (pH 7.2), and then mixed with 1× and 2× MIC peptides. A *P. aeruginosa* suspension at 2 × 10^5^ CFU/mL was prepared and added to the mixtures. The plate was incubated overnight at 37 °C, and bacteria growth was measured at 600 nm.

### Circular dichroism spectroscopy toward examining secondary structures

To investigate the secondary structure within different environments, 40 µM peptide solutions were prepared in 30 mM sodium dodecyl sulfate (SDS; Sigma-Aldrich), 50% trifluoroethanol (TFE; Sigma-Aldrich), or 10 mM sodium phosphate buffer (pH 7.2), with or without the addition of 0.1% LPS. Circular dichroism (CD) assays were then performed using a JASCO 810 spectropolarimeter (Jasco, Tokyo, Japan). CD spectra were recorded from 190 nm to 250 nm using a quartz cell with a 1.0 mm path length^60^.

### Large unilamellar vesicles preparation

Large unilamellar vesicles (LUVs) were prepared using the freeze-thaw method^61^. Briefly, we prepared the following lipid mixtures: phosphatidylethanolamine (PE) and polyethylene glycol (PG) at a 7:3 w/w ratio; phosphatidylcholine (PC), cholesterol (CH), and sphingomyelin (SM) at a 1:1:1 w/w ratio, and; PC:CH at a 2:1 w/w ratio. The mixtures were dissolved in chloroform, and then dried in argon gas. The remaining chloroform was removed by overnight lyophilization. The next day, dry lipid films were resuspended in 2.6 mL PBS by gently vortexing for 30 min. After nine freeze-thaw cycles under liquid nitrogen and a water bath at 50 °C, lipid suspensions were extruded 15–20 times through 0.2 µm polycarbonate membranes. Lipid concentration was determined with the phosphate assay, using a standard curve.

### Liposome aggregation and CD assay

Aggregation of LUVs were confirmed by measuring turbidity change. Peptides were prepared at concentrations ranging from 10 µM to 80 µM, and added to 400 µM LUV composed of PE:PG (7:3, w/w), PC:CH:SM (1:1:1, w/w), and PC:CH (2:1, w/w). Aggregation causes an increase in turbidity, which is quantified by measuring absorbance at 405 nm. The secondary structure was assessed using circular dichroism (CD) spectroscopy, as previously described^60^. CD spectra were measured in aqueous solution with 100 µM peptide in the presence of LUVs (400 µM).

### Calcein leakage assay

Peptide-induced membrane permeabilization was quantified by monitoring the calcein release from LUVs^62^. PC:PG liposome (9:1, w/w, mimicking a bacterial membrane surface) was prepared with 70 mM calcein as described above. The calcein-entrapped vesicles were removed by gel filtration chromatography on a Sephadex G-50 column. The calcein-loaded LUVs (10 µM) were mixed with different concentrations of peptides (0.00625–4 µM) in a 96-well black plate. The fluorescence generated from the released calcein was measured at an excitation wavelength of 480 nm and an emission wavelength of 520 nm.

### Outer membrane permeabilization assay

The outer membrane permeabilization activities of the peptides were determined using the NPN assay^63^. *P. aeruginosa* (ATCC 27853) was grown to mid-log phase at 37 °C, and harvested by centrifugation in 5 mM HEPES buffer (pH 7.2). Subsequently, the cell suspension (OD_600_ = 0.2) was mixed with 10 μM NPN in 96-well black plates. After addition of peptides at various final concentrations, or Triton X-100 at a final concentration of 0.1% (positive control), NPN fluorescence was measured (excitation wavelength of 350 nm, emission wavelength of 420 nm).

### Cytoplasmic membrane depolarization assay

The membrane depolarization activities of the peptides were determined using *P. aeruginosa* (ATCC 27853), and the membrane potential-sensitive fluorescent dye 3,3′-Dipropylthiadicarbocyanine iodide (DiSC_3_-5), as previously described^64^. Briefly, bacteria was washed in a buffer containing 5 mM HEPES (pH 7.3) and 20 mM glucose, and resuspended to an OD_600_ of 0.05 in a buffer containing 5 mM HEPES (pH 7.3), 20 mM glucose, and 0.1 M KCl. DiSC_3_-5 was then added at a final concentration of 1 µM. After 1 h incubation, mixture was transferred into 96-well plates and left until maximal dye uptake had been reached. Subsequently, peptides were added to the wells, and fluorescence intensity was monitored (excitation wavelength of 622 nm, emission wavelength of 670 nm).

### DNA binding assay

Gel retardation experiments were performed as described previously^28^. Briefly, 260 ng of plasmid DNA (pRSETB) was mixed with increasing amounts of peptides in a buffer containing 10 mM Tris-HCl (pH 8.0), 1 mM EDTA, 5% glucose, 20 mM KCl, and 50 µg/mL BSA. The following peptide/DNA ratios were used: 0.25:1, 0.5:1, 1:1, 1.5:1, 2:1, 3:1, and 4:1. Vials only containing DNA were used as negative controls. The mixtures were incubated for 10 min at 37 °C. Then, DNA sample buffer was added to each vial, and aliquots were separated by agarose gel electrophoresis (1% agarose, 100 V) in 0.5× TAE (Tris-acetate-EDTA) buffer. After ethidium bromide (EtBr) staining, DNA bands were visualized by UV illumination using a Bio-Rad Gel Documentation system (Hercules, CA, USA).

### Flow cytometry

*P. aeruginosa* (ATCC 27853) cells were washed three times with PBS buffer (pH 7.4), and resuspended in the same buffer to OD = 0.2. Bacteria were incubated for 1 h at 37 °C in the absence (negative controls) or presence of each tested peptide (at 2× MIC). Cells were centrifuged and then treated with propidium iodide (PI; Sigma-Aldrich) at a final concentration of 10 µg/mL for 30 min at 4 °C. After the incubation, cells were centrifuged at 10 000× *g* and resuspended in 1 mL PBS. Analysis was performed using a CytoFLEX flow cytometer (Beckman Coulter, Brea, CA, USA.).

### Efficacy of peptides in mice model of skin infection

BALB/c mice (6 weeks old) were used in this study. The mice were shaved and intradermally injected with 20 µl of *P. aeruginosa* (1 × 10^8^ CFU/ml) in PBS. After injection, the mice were treated with Hp1404-T1e and ciprofloxacin, and the mice were monitored daily. This study was carried out in strict accordance with the recommendations in the Guide for the Care and Use of Laboratory Animals of the National Institutes of Health, and approved by the Committee on the Ethics of Animal Experiments (CIACUC2017-S0042; Chosun University, Gwangju, South Korea).

## Electronic supplementary material

supplementary information
