# Supplementary material for: Mechanisms driving the antibacterial and antibiofilm properties of Hp1404 and its analogue peptides against multidrug-resistant Pseudomonas aeruginosa
Source: Sci Rep. 2018 Jan 29;8:1763. doi: 10.1038/s41598-018-19434-7 (PMC5789083; doi:10.1038/s41598-018-19434-7)
Supplement: Supplementary file 1 — supplementary information [file 41598_2018_19434_MOESM1_ESM.pdf]

Supplementary data

Mechanisms driving the antibacterial and antibiofilm properties of Hp1404 and its analogue peptides against multidrug-resistant *Pseudomonas aeruginosa*

Min Kyung Kim[1,2], Hee Kyoung Kang[2], Su Jin Ko[1,2], Min Ji Hong [1,2], Jeong Kyu Bang[3], Chang Ho Seo[4], Yoonkyung Park[1,2*]

[1] Research Center for proteineous Materials (RCPM), Chosun University, Kwangju, Republic of Korea

[2] Department of Biotechnology and BK21-Plus Research Team for Bioactive Control Technology, Chosun University, Kwangju, Republic of Korea

[3] Division of Magnetic Resonance, Korea Basic Science Institute, Ochang, Chung-Buk 363-883, Republic of Korea.

[4] Department of Bioinformatics, Kongju National University, Kongju 314-701, South Korea

*Corresponding author: Y. Park, Department of Biomedical Science, Chosun University, Gwangju 61452, Republic of Korea, Tel.: + 82 62 230 6854 (e-mail: y_k_park@chosun.ac.kr).

Materials and Methods

Peptide synthesis and purification. The peptides were synthesized using the solid-phase-9-fluorenylmethoxycarbonyl (Fmoc) method, as reported previously[1], on a Rink amide 4-methylbenzhydrylamine resin, using a Liberty microwave peptide synthesizer (CEM, Matthews, NY, USA). The following chemicals were used as linkage reagents: 0.45 M 2-(1H-benzotriazole-1-yil)-1,1,3,3-tetramethyluronium hexafluorophosphate (HBTU) diluted in dimethylformamide (DMF), 0.1 M *N*-hydroxybenzotriazole (HOBt) diluted in piperidine/DMF, and 2 M *N*,*N*-diisopropylethylamine (DIEA) diluted in *N*-methylpyrrolidone (NMP). After washing with dichloromethane (DCM), cleavage was performed by incubating for 2 h at 25 °C in a trifluoroacetic acid (TFA) solution containing water, phenol, and triisopropylsilane. The crude peptide was precipitated by dilution with ice-cold diethyl ether, then spread on the tube wall, and dried. After resuspension in 25 °C water, the peptide was purified by reversed phase high-performance liquid chromatography (RP-HPLC) on a Jupiter C18 column (4.6 × 250 mm, 300 Å, 5 µm; Phenomenex, Torrance, CA, USA). The molecular weights of the synthetic peptides were confirmed through matrix-assisted laser desorption ionization-time of flight (MALDI-TOF) mass spectrometry. The peptides were dissolved in deionized water (DI H_2O) and solutions were stored at −20 °C. In this study, all used peptides were > 95 % pure.

Time-killing assay. Mid-log phase grown *P. aeruginosa* ATCC 27853 (2×10^5 CFU/mL) were treated with 2× MIC of synthetic peptides. For each hour, samples were diluted and plated in triplicate on NB agar plates. After an 18-h incubation, the colonies were counted and average counts were determined.

Calcein leakage assay. LUVs were prepared as described above[2], two types of liposome were prepared: PC:CH:SM (1:1:1, w/w, mimicking mammalian cell membranes). Dried lipids were mixed and vortexed with the dye buffer solution (70 mM calcein, HEPES, pH 7.3). The suspension was freeze-thawed in liquid nitrogen for nine cycles, after which, the calcein-entrapped vesicles were removed by gel filtration chromatography on a Sephadex G-50 column. The calcein-loaded LUVs (10 µM) were mixed with different concentrations of peptides (0.00625 - 4 µM) in a 96-well black plate. The fluorescence generated from the

released calcein was measured at an excitation wavelength of 480 nm and an emission wavelength of 520 nm.

Trypsin stability assay. Peptides were treated with trypsin, and results were evaluated using RP-HPLC, as described previously[3]. Briefly, 20 µg of peptide was mixed with 100 µL of 10 mM sodium phosphate buffer (pH 7.4) containing 25 nM trypsin (Sigma-Aldrich), and incubated at 37 °C for 1 h or 2 h. After incubation, 20 µL of each sample was analysed by RT-HPLC on a Jupiter C18 column (4.6 × 250 mm, 300 Å, 5 µm; Phenomenex).

PI uptake assay. Bacterial membrane damage was assessed through monitoring the changes in fluorescence generated by the propidium iodide (PI) dye, as described previously[4]. Briefly, *P. aeruginosa* ATCC 27853 was grown in NB to logarithmic phase of growth, and then diluted to OD_{600} = 0.25 in 10 mM sodium phosphate (pH 7.2) buffer with 10 % medium. PI was mixed with bacteria to a final concentration of 20 µM. Aliquots of 200 µL were loaded into 96-well black plates and subsequently treated with peptides (at 1×, 2×, or 4× MIC) and ciprofloxacin. For the control, 10 mM sodium phosphate buffer was used. PI fluorescence was monitored at an excitation wavelength of 580 nm and emission wavelength of 620 nm, using a microplate reader (Molecular Devices Co., Sunnyvale, CA, USA), during 30 min.

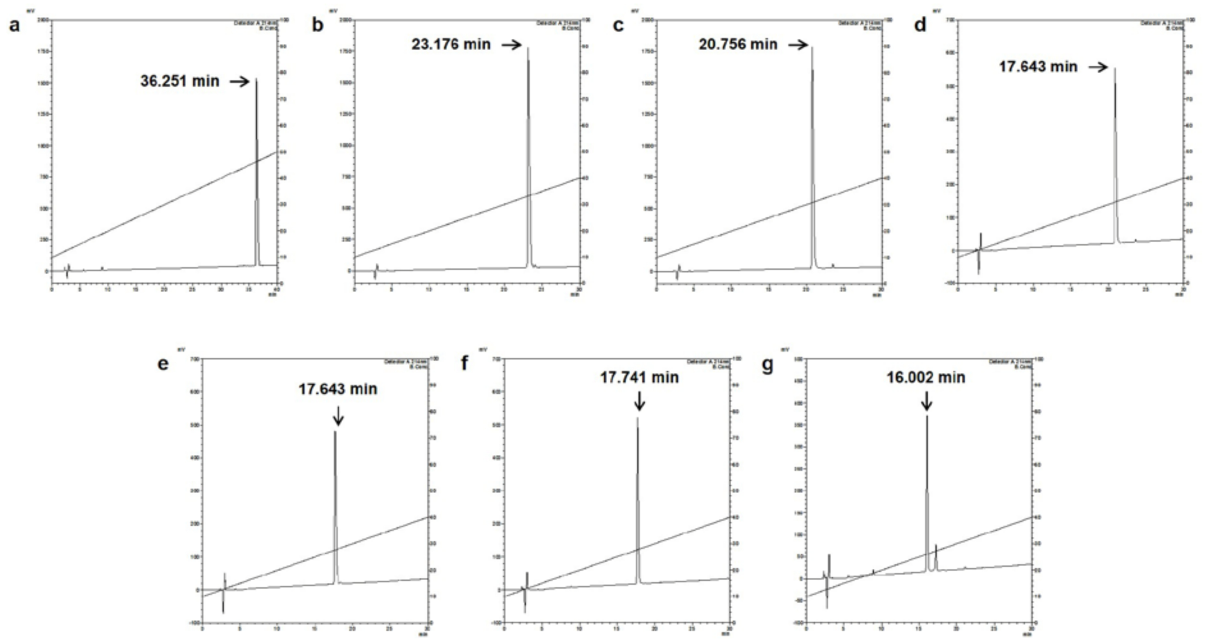

Figure S1: RP-HPLC profile on C18 columns as measured at 214 nm. (a) Hp1404, (b) Hp1404-T1, (c) Hp1404-T1a, (d) Hp1404-T1b, (e) Hp1404-T1c, (f) Hp1404-T1d, and (g) Hp1404-T1e. Black arrows indicate peptide retention time.

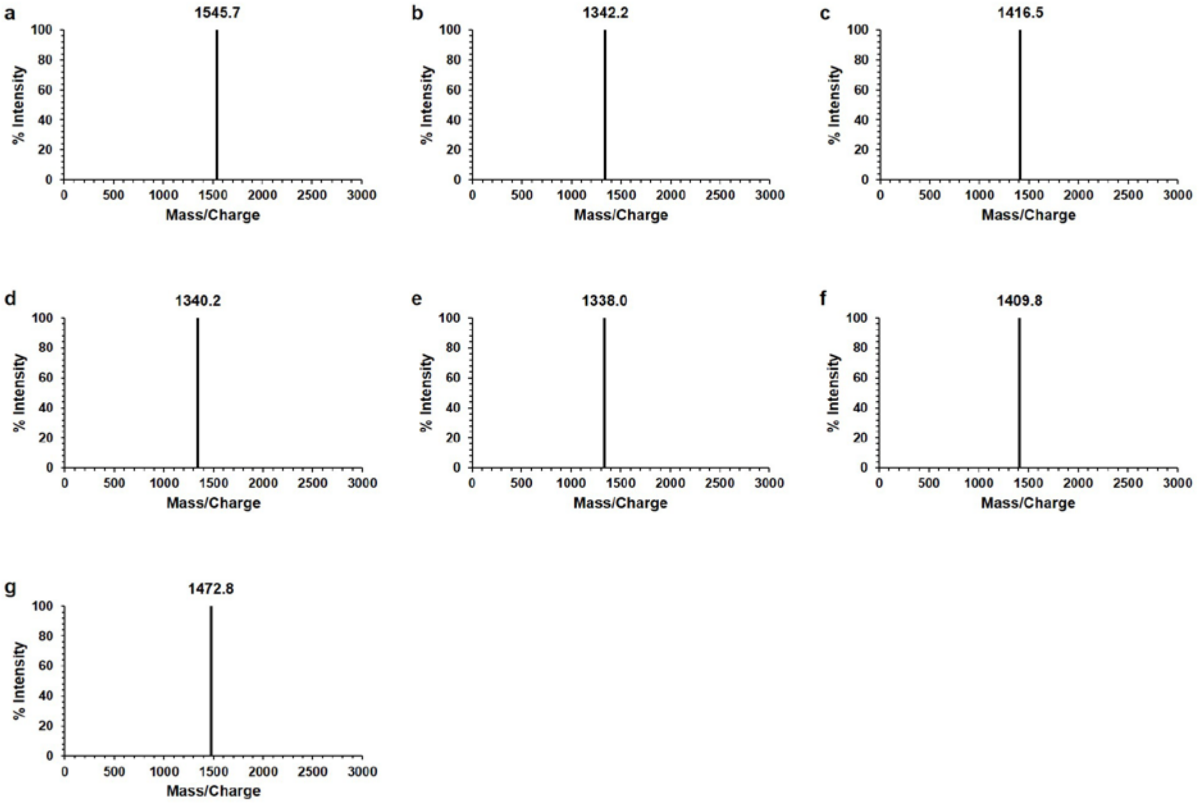

Figure S2. MALDI mass spectrometric analysis of peptides. (a) Hp1404, (b) Hp1404-T1, (c) Hp1404-T1a, (d) Hp1404-T1b, (e) Hp1404-T1c, (f) Hp1404-T1d, and (g) Hp1404-T1e. The respective Mass/Charge ratios were 1545.7, 1342.2, 1413.5, 1340.2, 1338, 1409.8, and 1472.8.

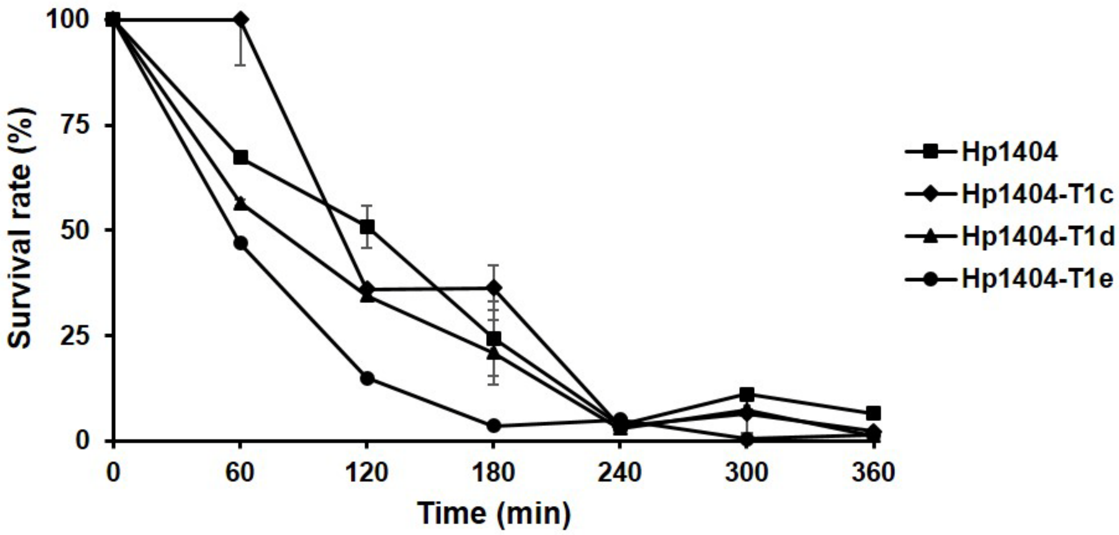

Figure S3. Time-kill-kinetics of synthetic peptides against *P. aeruginosa* ATCC 27853. The bacteria killing capacity of each peptide was observed for 6 hours after its injection.

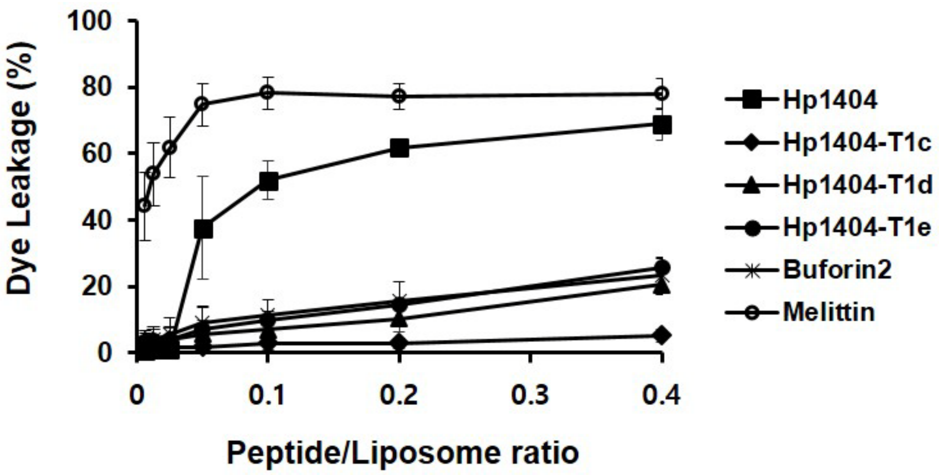

Figure S4. Calcein leakage assay. Percent of calcein leakage from PC:CH:SM (1:1:1, w/w ratio) vesicle.

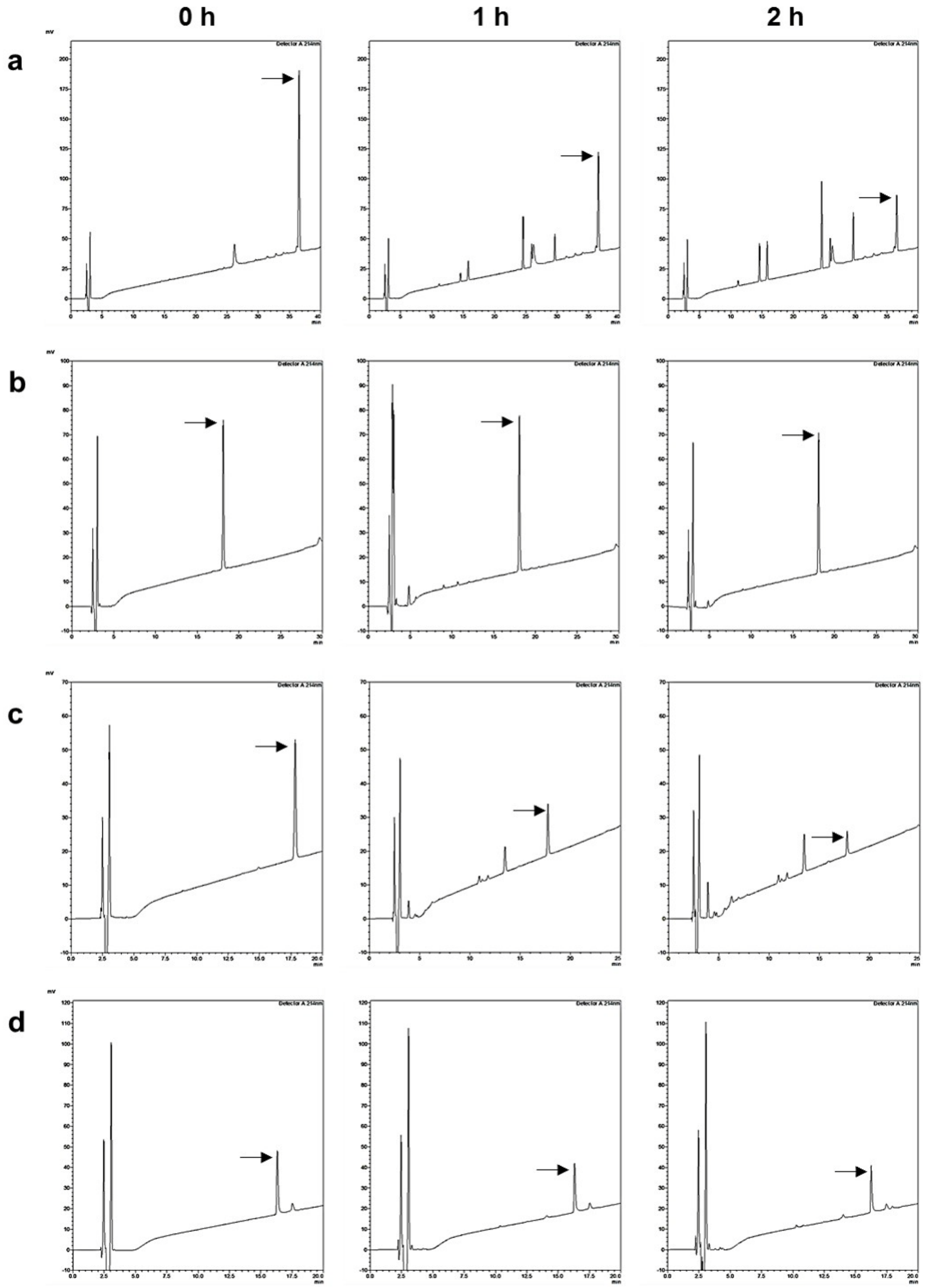

Figure S5. RP-HPLC chromatograms (C_{18} columns; detector wavelength = 214 nm) of (a) Hp1404, (b) Hp1404-T1c, (c) Hp1404-T1d, and (d) Hp1404-T1e samples before trypsin treatment, as well as after 1 and 2 h of such treatment.

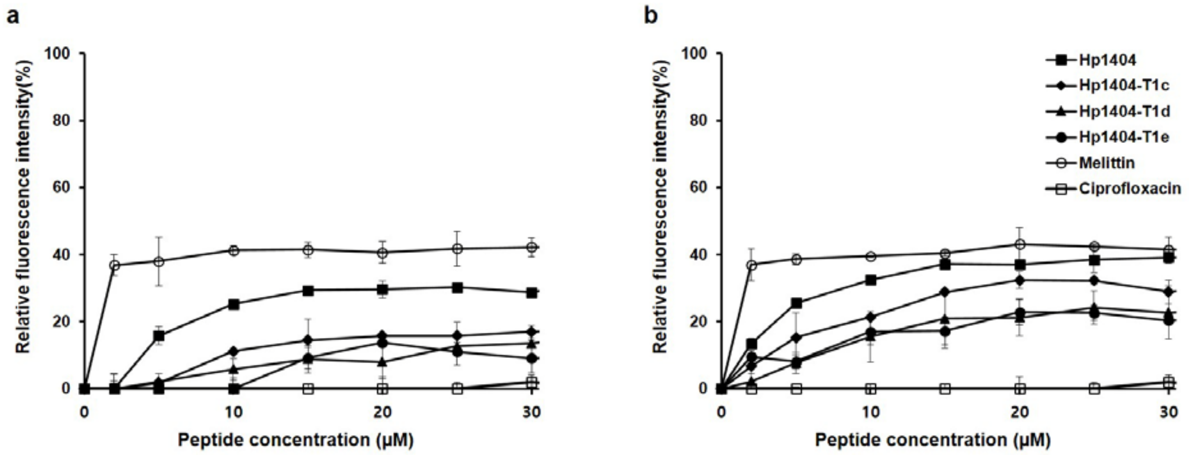

Figure S6. PI uptake assay. Permeabilization of the cytoplasmic membrane of synthetic peptides was indicated by the percentage of propidium iodide fluorescence for 30 min exposure. (a) 1X MIC. (b) 2X MIC.

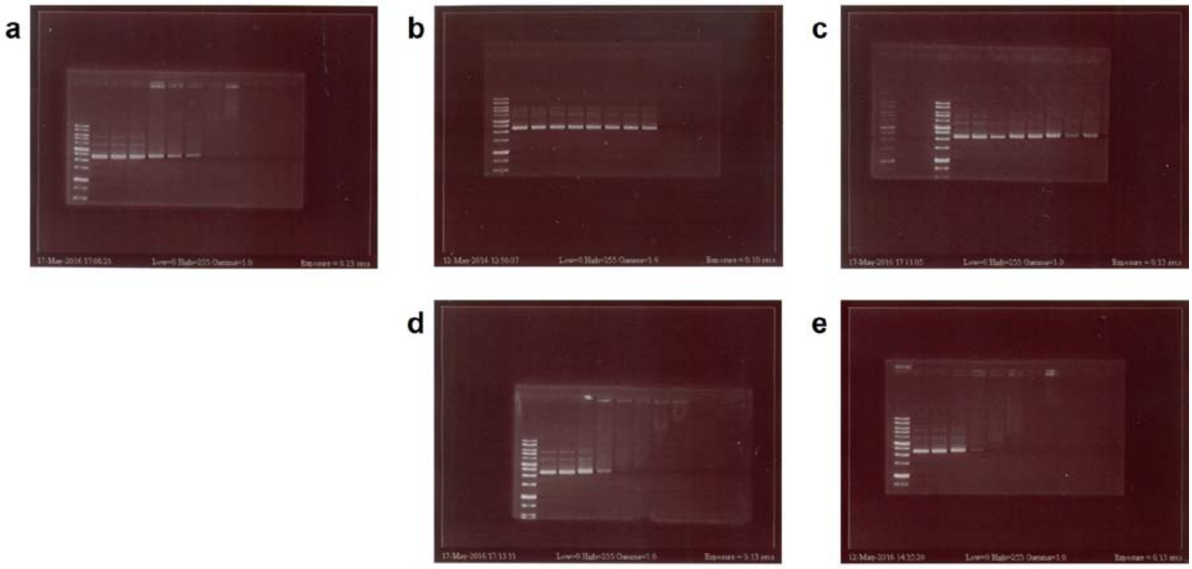

Figure S7. DNA binding assay. (a) Buforin 2, (b) Hp1404, (c) Hp1404-T1c, (d) Hp1404-T1d, or (e) Hp1404-T1e.

Supplemental References

1 Fields, G. B. & Noble, R. L. Solid phase peptide synthesis utilizing 9-fluorenylmethoxycarbonyl amino acids. *Int J Pept Protein Res* **35**, 161-214 (1990).

2 Gopal, R., Seo, C. H., Song, P. I. & Park, Y. Effect of repetitive lysine-tryptophan motifs on the bactericidal activity of antimicrobial peptides. *Amino Acids* **44**, 645-660, doi:10.1007/s00726-012-1388-6 (2013).

3 Shin, Y. P. *et al.* Antimicrobial activity of a halocidin-derived peptide resistant to attacks by proteases. *Antimicrob Agents Chemother* **54**, 2855-2866, doi:10.1128/aac.01790-09 (2010).

4 Mohamed, M. F., Hammac, G. K., Guptill, L. & Seleem, M. N. Antibacterial activity of novel cationic peptides against clinical isolates of multi-drug resistant Staphylococcus pseudintermedius from infected dogs. *PLoS One* **9**, e116259, doi:10.1371/journal.pone.0116259 (2014).
